# Topological Defects in Topological Insulators and Bound States at Topological Superconductor Vortices

**DOI:** 10.3390/ma7031652

**Published:** 2014-03-04

**Authors:** Vincenzo Parente, Gabriele Campagnano, Domenico Giuliano, Arturo Tagliacozzo, Francisco Guinea

**Affiliations:** 1 Dipartimento di Fisica, Università di Napoli Federico II, Via Cintia, Napoli 80126, Italy; E-Mails: proteo855@gmail.com (V.P.); g.campagnano@gmail.com (G.C.); 2 Consiglio Nazionale delle Ricerche - Superconductors, Oxides and other Innovative Materials and Devices (CNR-SPIN), Via Cintia, Napoli 80126, Italy; 3 Dipartimento di Fisica, Università della Calabria Arcavacata di Rende, Cosenza I-87036, Italy; E-Mail: domenico.giuliano@fis.unical.it; 4 Istituto Nazionale Fisica Nucleare, Gruppo Collegato di Cosenza, Arcavacata di Rende, Cosenza I-87036, Italy; 5 Instituto de Ciencia de Materiales de Madrid, Consejo Superior Investigación Cientifica (ICMM-CSIC) , Cantoblanco, Cale Sor Juana Ines de la Cruz 3, Madrid 28049, Spain; E-Mail: paco.guinea@icmm.csic.es

**Keywords:** Dirac electrons, topological defects, two-band topological insulators, Majorana bound state

## Abstract

The scattering of Dirac electrons by topological defects could be one of the most relevant sources of resistance in graphene and at the boundary surfaces of a three-dimensional topological insulator (3D TI). In the long wavelength, continuous limit of the Dirac equation, the topological defect can be described as a distortion of the metric in curved space, which can be accounted for by a rotation of the Gamma matrices and by a spin connection inherited with the curvature. These features modify the scattering properties of the carriers. We discuss the self-energy of defect formation with this approach and the electron cross-section for intra-valley scattering at an edge dislocation in graphene, including corrections coming from the local stress. The cross-section contribution to the resistivity, *ρ*, is derived within the Boltzmann theory of transport. On the same lines, we discuss the scattering of a screw dislocation in a two-band 3D TI, like Bi_1−_*_x_*Sb*_x_*, and we present the analytical simplified form of the wavefunction for gapless helical states bound at the defect. When a 3D TI is sandwiched between two even-parity superconductors, Dirac boundary states acquire superconductive correlations by proximity. In the presence of a magnetic vortex piercing the heterostructure, two Majorana states are localized at the two interfaces and bound to the vortex core. They have a half integer total angular momentum each, to match with the unitary orbital angular momentum of the vortex charge.

## 1. Introduction

Boundaries in a topological insulator (TI) host Dirac electrons propagating with a linear dispersion in energy [1]. On the other hand, it was recognized long ago that the low energy electronic properties of a graphene sheet, which is a weak topological material, is a semimetal and can be described close to the neutrality point with the Dirac Hamiltonian [2].

In the recent past, the charge carrier mobility in a single graphene layer has been extensively investigated [3,4]. Graphene resistivity was experimentally found to be inversely proportional to concentration *n* of the charge carriers, which means that their mobility is almost independent of *n* [5–7]. This uncommon behavior cannot be attributed to short-range potentials, due to defects on the scale of the lattice parameter, *a*. Indeed, these defects are believed to provide only a small additional contribution to the resistivity proportional to the impurity concentration [8,9]. Instead, the contribution due to charged impurities, acting as long-range Coulomb scatterers [10], seems to fit with the experimental finding in the case of graphene sheets attached to substrates [8,11–13]. In the case of suspended graphene [14], the matter is still unsettled and has motivated an extended search for other scattering mechanisms. Particularly, corrugations and ripples have been found, and the systematic investigation of their effect on the electrical and optical properties are far from being clarified [3,15–18]. Suspended graphene should not be so influenced by the charge impurities, except for, possibly, trapped clusters in the wake of the corrugations [19,20]. Other sources of scattering, topological point-like lattice defects, can induce only little stretching locally, but contribute significantly to the reduction of the mobility. We find that this is the case of an edge dislocation centered on a pentagon-heptagon defect, which does not generate any curvature in the graphene sheet. Furthermore, wedge disclinations due to isolated pentagons or heptagons in the lattice structure could be considered, although they are believed to cost higher formation energy.

On the other hand, the analysis of the conductance properties of boundary states at the surface of a three-dimensional (3D) TI, like Bi_2_Se_3_, Bi_2_Te_3_ and Bi_1−_*_x_*Sb*_x_*, is still in its infancy. Experimentally, it is difficult to tune the Fermi energy within the gap of the 3D TI to study the mobility of the Dirac states located at the boundaries. Besides, in most cases, mesoscopic samples, like TI flakes or TI nanowires, are plagued by impurity bands within the insulating gap. Therefore, it is difficult to isolate the contribution of the Dirac electrons from the bulk contribution [21–25]. Measures of the mobility as derived from Shubnikov–de Haas oscillations or Hall resistance give different results. Aharonov–Bohm oscillations of

Dirac electrons at the surface of nanowires have been measured [26–28]. To the best of our knowledge, the influence of topological defects, such as the screw dislocations or the wedge disclinations on the transport properties, has not been investigated yet.

In a previous paper [29], we considered the contributions to the resistivity, due to elastic scattering by a smooth curved bump at the surface of a 3D TI [30]. We considered the unrelaxed situation and attacked the problem directly in the continuum low energy limit, by introducing the defect as a change of the metric experienced by the electrons [18,31]. In the unrelaxed lattice, the local Lorentz invariance is assumed to be still conserved. The defect itself is effectively described as an Aharonov–Bohm flux pinned at its center.

In the case of graphene, we see that the contribution of a collection of isolated edge dislocation to resistivity in the Boltzmann semiclassical limit is found to be ∝ 1/*n*. Scattering of an edge dislocation also depends on the orientation of the Burgers vector and, therefore, on the angle of the incoming electron wave, but this feature does not introduce any additional *k* dependence, if some averaging over randomness is implied. Besides, the resistivity of the edge dislocation is found to be finite, close to the neutrality point. We find that any additional contribution to the resistivity due to weak local strain vanishes close to the neutrality point with positive powers of *k_F_*. Here, *k_F_* is the Fermi wavevector and *ℓ* is the size of the perturbation, which is much larger than the lattice spacing, *a*. Although the graphene monolayer is characterized by two Dirac cones centered at the two valley points, *K* and $\bar{K}=4\pi /3a(0,\pm 1)$, in the Brillouin zone [32], elastic scattering potentials, smooth on the lattice scale, such as ripples or point-like defects, are likely not to change the isospin and the valley of the scattering electrons, nor their energy spectrum drastically, unless the strain produces gaps or zero energy states [20,33]. We point out that intravalley scattering produced by edge dislocations can give a relevant contribution to the resistivity, especially in the absence of sizable scattering by charges or resonant impurities. The question arises: how large is their formation energy in graphene? We will address this point in Section 3.3.

Similarly, the presence of screw dislocations of Burgers vector $\vec{b}$ can be envisaged in a 3D TI. In the present work, we apply the analytical methods used to discuss an edge dislocation in the graphene sheet to a screw dislocation in a two-band 3D TI. In this case, helical bound states can propagate along its axis. The Hamiltonian can be expanded linearly in $\vec{k}$ close to a time reversal invariant momentum (TRIM), M*_ν_*. Two gapless modes of opposite helicity are present, provided the constraint, $M_{\nu }\cdot \vec{b}$, is satisfied [34]. The reference case is the alloy, Bi_1−_*_x_*Sb*_x_*, while TIs like Bi_2_Se_3_ and Bi_2_Te_3_ cannot fulfill the constraint, because their 3D Dirac point is located at Γ. We exhibit the wavefunctions of the bound states and their properties in Section 5.

Superconductive proximity induced at an interface between a 3D TI and a conventional superconductor has been attracting a lot of interest recently, due to the expectation that Majorana bound states (MBS) could exist under appropriate conditions [35–37]. In the presence of a magnetic field, the screw dislocation could host a vortex line with little energy cost. A vortex line piercing a 3D TI sandwiched between two even-parity superconductors could bind two Majorana quasiparticle excitations at the opposite interfaces. After the pioneering work of Fu and Kane, this feature has been discussed by various authors [38–40] in great detail. Some of us, by solving the Bogolubov–de Gennes equations analytically in the long wavelength limit, exhibited the wavefunctions of the MBS in the two-band 3D TI model and discussed how the MBS depends on the parity of the order parameter of the superconductor inducing the proximity [41]. These results are briefly summarized in Section 6. When proximity involves odd-parity pairing, the modes appearing in the superconducting gap opened in the Dirac dispersion are charged surface Andreev bound states. They originate from interfacial circular states of definite chirality, centered at the vortex singularity and decay with damped oscillations away from the interfaces of the TI film. The case of d-wave-induced pairing in quasi-one-dimensional TI nanostructures was also discussed [42,43].

In Section 2, we sketch our approach by introducing some generalities about the change of coordinates in the presence of defects and curvature, to make the paper self contained [44]. In Section 3, we discuss the edge dislocation in a graphene sheet and compare the contributions to the cross-section coming from the topological defect when the lattice is unrelaxed with the one due to the stress-induced relaxation. The latter turns out to be negligible with respect to the former in the low electron density limit. A detailed account of the derivation of the phase shifts for the scattering of an Aharonov–Bohm (A–B) flux can be found in Appendix A. The contribution to the resistivity is also calculated, in the relaxation time approximation, as well as the self-energy of the defect, which requires Green’s function, which is derived in Appendix B. In Section 4, we introduce a two-band model for the 3D TI and refer to Appendix C for the calculation of the wavefunctions of the Dirac dispersed states localized at the boundary. Section 7 concludes the paper with a summary and few final remarks.

## 2. Dirac Electrons on a Free Surface in the Long Wavelength Limit

The long wavelength dynamics of Dirac electrons propagating on a flat two-dimensional boundary at energies close to the neutrality point of the Dirac cone is described by the Dirac equation:

$$\gamma^{a}\partial_{a}\Psi =0$$ (1)

where *a* = 0,1,2, and the Dirac matrices are γ^0^ = *−iσ*_z_, γ^1^ = *σ_y_*, γ^2^ = −*σ_x_*. In this Section, we consider the generalization of the algebra of the Dirac matrices describing a flat surface, to include topological defects and possible curvatures, in the absence of strain. According to the Equivalence Principle [18], given a frame to be referred to as the “curved frame”; from now on, it is possible to introduce a local flat, *x^a^*, frame at each point. The components of the Jacobian matrix for the transformation from the coordinates, *x^μ^*, defined on the whole manifold and the local parametrization, are the tetrads, *e^a^* *_μ_*:

$$e^{a}{}_{\mu }=\frac{\partial x^{a}}{\partial x^{\mu }}$$ (2)

The inverse of the tetrads, *e_a_* *^μ^*, are defined through the orthogonality relation *e_a_* *^μ^* *e^a^* *_ν_* *=* *δ^μ^* *_ν_*. Our aim is to substitute the Minkowski metric, *η_ab_*, of the flat space with the metric, *g_µν_*, which is singular in the case of a topological defect. We follow the convention to use Latin letters and overlined numbers, *a*, *b*, …, 1̄,2̄, to refer to the local frame, as opposed to the Greek letters, which are refer to the curved frame. The tetrads satisfy the completeness relation, *e^a^* *_μ_* *e_b_* *^μ^* *= δ^a^* *_b_*. They are linked to the metric according to:

$$g_{\mu \nu }=\eta_{ab}e^{a}{}_{\mu }e^{b}{}_{\nu }$$ (3)

The Dirac matrices γ*^μ^* = *e_a_* *^μ^*γ*^a^* satisfy the anticommutation relation:

$$\{\gamma^{\mu },\gamma^{\nu }\}=2g_{\mu \nu }$$ (4)

On formulating the Lorentz covariance of the Dirac equation locally, we replace the derivatives with the covariant derivatives:

$$\nabla_{\mu }=\partial_{\mu }+\Gamma_{\mu }=\partial_{\mu }+\frac{i}{2}\Gamma^{a}{{}_{\mu }}^{b}\sum_{ab}$$ (5)

where ∑*_ab_* are the generators of the spinorial representation of the Lorentz group and are expressed in terms of the commutators as ∑*_ab_* = *i*/2[γ*_a_*, γ*_b_*]. The connection coefficients, Γ*^a^* *_μ_* *^b^*, are given by Γ*^a^* *_μ_* *^b^* *= e^a^* *_ν_∂_μ_e^b^^ν^*. The massless Dirac equation on curved space then reads:

$$\gamma^{\mu }\nabla_{\mu }\Psi =0$$ (6)

Making explicit the rotation of the Dirac matrices and the covariant derivatives, the stationary part of Dirac equation on curved space time is:

$$-i\hbar \upsilon_{F}\sigma^{a}e_{a}{}^{\mu }(\partial_{\mu }+\Gamma_{\mu })\Phi =E\Phi$$ (7)

In full generality, defining the components of the affine connection as Γ*_λη_* *^μ^* = (*∂_λ_e^a^_η_*) *e_a_^μ^*, the components of the torsion are:

$$T_{\lambda \eta }{}^{\mu }=\frac{1}{2}(\Gamma_{\lambda \eta }{}^{\mu }-\Gamma_{\eta \lambda }{}^{\mu })=\frac{1}{2}(\partial_{\text{λ}}e^{a}{}_{\eta }-\partial_{\eta }e^{a}{}_{\text{λ}})e_{a}{}^{\mu }$$ (8)

while the Riemann tensor is defined as:

$$R_{\mu \nu \lambda }{}^{\kappa }=\partial_{\mu }\Gamma_{\nu \lambda }{}^{\kappa }-\partial_{\nu }\Gamma_{\mu \lambda }{}^{\kappa }-\Gamma_{\mu \lambda }{}^{\sigma }\Gamma_{\nu \sigma }{}^{\kappa }+\Gamma_{\nu \lambda }{}^{\sigma }\Gamma_{\mu \sigma }{}^{\kappa }$$ (9)

Equation (8) is clearly zero if the tetrads are regular functions, *i.e*., if they have continuous second derivatives, due to the Schwartz lemma. If the Riemann tensor also vanishes, the transformation, *x^μ^*(*x^a^*), is just a change of coordinates of the flat space. Tetrads that are not regular could give curvature, torsion or both. An unrelaxed topological defect does not introduce any curvature, so that only the rotation of the Dirac matrices has to be accounted for.

## 3. The Edge Dislocation in Graphene

**Figure 1.**
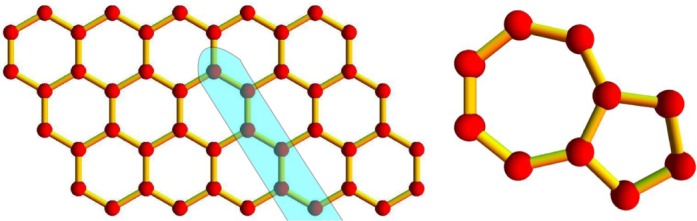
Edge dislocation appearing as a pentagon-heptagon pair in the perfect lattice of a graphene monolayer.

An edge dislocation in the graphene sheet, centered on the origin, is obtained by cutting the plane; let us say in correspondence of the negative *x*_1_ half axis and by adding a half line of carbon atoms [45]. In the case of a hexagonal lattice, the line of atoms could be interpreted as an armchair row. In fact, the whole honeycomb lattice could be recovered by arranging arrays of armchair rows in a square structure. The dislocation produces a pentagon-heptagon pair in the origin of the frame, as shown in Figure 1. In the unrelaxed structure, the other hexagons are not affected. This kind of defect does not mix the valleys. Smooth potentials are likely not to change the isospin and the valley of the scattering electrons, nor their energy spectrum, drastically, unless the strain produces gaps or zero energy states [4,33].

Let $\vec{b}$ be the Burgers vector pointing down along the *x*_2_ –axis, orthogonal to the negative *x*_1_-axis. The Burgers vector defines a singular coordinate transformation:

$$\begin{array}{cc} x^{\bar{1}}=x^{1}, & x^{\bar{2}}=x^{2}-\frac{b}{2\pi } \end{array}\arctan\frac{x^{2}}{x^{1}}$$ (10)

where the branch cut of the inverse tangent is on the negative *x*_1_-axis. The tetrads are easily derived from the infinitesimal transformation *dx^a^* *= e^a^* *_μ_dx^μ^*, thus obtaining:

$$e^{a}{}_{\mu }=\left(\;\begin{array}{cc} \begin{array}{c} 1\\ \frac{b}{2\pi }\frac{y}{x_{1}^{2}+x_{2}^{2}} \end{array} & \begin{array}{c} 0\\ 1-\frac{b}{2\pi }\frac{x_{1}}{x_{1}^{2}+x_{2}^{2}} \end{array} \end{array}\right)$$ (11)

Equation (11), as such, describes an unrelaxed configuration. The equilibrium configuration could be restored by adding an effective gauge potential to the Dirac equation, which may further change the spin connection, without influencing, however, the holonomy on the wave function, since the elastic deformation does not add any curvature. This is a way of restating the Saint Venant conditions for the two-dimensional case.

The Dirac matrices γ*^μ^* *= e_a_* *^μ^*γ*^a^* can be easily found by inversion of the tetrads:

$$\begin{array}{c} \gamma^{1}=\sigma_{1}\\ \gamma^{2}=\frac{1}{1-\frac{b}{2\pi }\frac{x_{1}}{x_{1}^{2}+x_{2}^{2}}}\left[-\frac{b}{2\pi }\frac{x_{2}}{x_{1}^{2}+x_{2}^{2}}\sigma_{1}+\sigma_{2}\right] \end{array}$$ (12)

In the case of an edge dislocation, the Riemann tensor generated by these tetrads, *R_μνλ_* *^κ^*, vanishes [31], so that the connection on an edge dislocation can be put to zero. On the contrary, the torsion is *δ* – like, as it encodes the mismatch in the parallel transport occurring at the topological defect [46]:

$$\begin{array}{cc} T_{12}{}^{\bar{1}}=-b\delta (\vec{r}), & T_{12}{}^{\bar{2}}=0 \end{array}$$ (13)

Since the connection vanishes, the spin connection is also zero. Therefore, only the rotation of Dirac matrices appears in the Dirac equation.

Let $\vec{k}=\vec{p}-\vec{K}$ be the component of the wavevector referring to the valley point, $\vec{K}$, and *θ_k_* the angle of the local momentum, $\vec{k}$. If $\Phi_{s}^{0}(\vec{k})$ is a spinor satisfying the equation $\gamma^{a}k_{a}\Phi_{s}^{0}=(s=\pm )$:

$$\Phi_{s}^{0}\left(\vec{k}\right)=\frac{1}{\sqrt{2}}\left(\begin{array}{c} 1\\ s\;e^{i\theta \kappa } \end{array}\right)$$ (14)

the solution of the rotated Dirac equation can be written down in full generality as (*μ* = 0, 1, 2) [47]:

$$\Psi_{s}(\vec{r})=\Phi_{s}^{0}(\vec{k})\;e^{-i\vec{K}\cdot \vec{r}}\exp i\int_{Cr}p_{\mu }(r)dx^{\mu }/\hbar$$ (15)

Here, the integral is to be performed along the geodesic path, *C_r_*, connecting a reference point, ${\vec{r}}_{0}$, with the actual point, $\vec{r}$. The linear transformation *e^a^* *_μ_k_a_* *= k_μ_* defines *k_μ_*(**r**).

This is indeed a solution, because, by substituting the spinor of Equation (15) in the Dirac equation, we get:

$$\gamma^{\mu }\partial_{\mu }\Psi_{s}=\gamma^{a}e_{a}{}^{\mu }k_{\mu }(r)\Psi_{s}\equiv \gamma^{a}e_{a}{}^{\mu }e^{b}{}_{\mu }k_{b}\Psi_{s}=0$$ (16)

where the equality to zero stems from the definition of Φ_0_ given in Equation (14).

Equation (15) is a general way to take account of the rotation of the Dirac matrices, and it provides the full solution when no spin connection is present. In the case of the edge dislocation, the differential form appearing in the phase of Equation (15), when calculated using the tetrads of Equation (11), is:

$$k_{\mu }(x)dx^{\mu }=\left(k_{\bar{1}}+\frac{k_{\bar{2}}b}{2\pi }\frac{x_{\bar{2}}}{x_{\bar{1}}{}^{2}+x_{\bar{2}}{}^{2}}\right)dx^{\bar{1}}+\left(k_{\bar{2}}-\frac{k_{\bar{2}}b}{2\pi }\frac{x_{\bar{1}}}{x_{\bar{1}}{}^{2}+x_{\bar{2}}{}^{2}}\right)dx^{\bar{2}}$$ (17)

The quantities, $k_{\bar{1},\bar{2}}$, represent the components of the momentum in the local inertial frame. The curl of this differential form is zero. Indeed, it is the differential of the accumulated phase given by:

$$\vec{k}\cdot \vec{r}-\frac{k_{\bar{2}}b}{2\pi }\arctan\frac{x_{\bar{2}}}{x_{\bar{1}}}$$ (18)

It follows that the integral in Equation (15) is independent of the path, and it is readily calculated. The full spinor solution for electrons having a $\vec{p}$ vector close to the valley point, $\vec{K}$, includes a fast oscillating plane wave prefactor, $e^{i\vec{K}\cdot \vec{r}}$, giving:

$$\Psi_{\vec{p},s}(\vec{r})=\frac{1}{\sqrt{2}}\left(\begin{array}{c} 1\\ s\;e^{i\theta k} \end{array}\right)e^{i\vec{p}\cdot \vec{r}}\;e^{-i\vec{k}\cdot \frac{\vec{b}}{2\pi }\theta r}$$ (19)

The edge dislocation produces a vortex-like singularity in the graphene sheet, unless $\vec{k}∥\hat{x}$, that is, unless the propagation is along the branch cut. The solution corresponds to a plane wave scattering of a flux line of flux $\vec{k}\cdot \vec{b}$, piercing the sheet at the origin, and acquiring an Aharonov–Bohm phase in circulation. The single valuedness of the wavefunction in circulating around the dislocation and the *C*_3_ rotational symmetry of the graphene lattice requires that $\vec{k}\cdot \vec{b}=2\pi /3$.

### 3.1. Cross-Section of the Unrelaxed Topological Defect

The phase shifts of a particle incoming with momentum $\vec{p}$ scattered with angular momentum quantum number *m* by the edge dislocation, $\delta_{m}(\vec{p})$, can be easily derived [48,49]. They are (see Appendix A for the details):

$$\delta_{m}(\vec{p})=-\frac{\pi }{2}\left(\left|m+\frac{\vec{k}\cdot \vec{b}}{2\pi }\right|-\left|m\right|\right)$$ (20)

Defining the outgoing wave (*ϕ* *≡* *θ_r_* *−* *θ_k_*):

$$\psi_{\mathrm{out}}(\vec{r})∼\left(\begin{array}{c} 1\\ se^{i\theta k} \end{array}\right)\left[e^{i\vec{k}\cdot \vec{r}}+f(\phi ,\vec{p})\frac{e^{ikr}}{\sqrt{r}}\right]$$ (21)

the scattering amplitude, $f(\phi ,\vec{p})$, is:

$$f(\phi ,\vec{p})=\sqrt{\frac{1}{2\pi k}}\sum_{m=-\infty }^{\infty }{\left(-1\right)}^{m}[e^{2i\delta_{m}(\vec{p})}-1]e^{im\phi }$$ (22)

The total cross-section, with $f(\phi ,\vec{p})$ given by Equation (22), is:

$$\sigma_{\mathrm{disl}}^{\mathrm{topological}}=\int d\phi {\left|f(\phi ,\vec{p})\right|}^{2}=\frac{4}{k}\sum_{m=-\infty }^{\infty }\sin^{2}\delta_{m}(\vec{p})$$ (23)

Close to the neutrality point (limit $\vec{k}\to 0$):

$$\sigma_{\mathrm{disl}}^{\mathrm{topological}}∼{\left(k_{F}R\right)}^{-2}$$ (24)

The resistivity, *ρ*, can be estimated with the Boltzmann relaxation-time $\left(\tau \left(k_{F}\right)\right)$ approximation as:

$$\rho (k_{F})=\frac{2}{e^{2}\upsilon_{F}^{2}\nu (0)}\frac{1}{\tau (k_{F})}$$ (25)

where $\nu (0)=k_{F}/\left(\pi \hbar \upsilon_{F}\right)$ is the density of states at the Fermi level for double spin, but one valley The usual definition of the total relaxation rate is:

$$\frac{1}{\tau (k_{F})}=\frac{2\pi }{\hbar }\nu (0)\int d\epsilon_{p^{\prime }}\delta (\epsilon_{F}-\epsilon_{p^{\prime }})\int_{0}^{2\pi }d\theta_{\vec{k}-{\vec{k}}^{\prime }}\left(1-\hat{k}\cdot {\hat{k}}^{\prime }\right)|<k|t^{\mathrm{eff}}|k^{\prime }>{|}^{2}$$ (26)

The relaxation rate, related to the imaginary part of the self-energy, is expressed in terms of the *t* – matrix element < *k*′|*t^e f f^*|*k* > for an outgoing circular wave, of incoming wavevector $\vec{k}+\vec{K}$ at the Fermi energy, scattered elastically into a plane wave of wavevector $\vec{p}={\vec{k}}^{\prime }+\vec{K}$ by the extra potential arising in Equation (7), of scattering amplitude $f(\phi ,\vec{p})$. It is:

$$\begin{array}{l} \langle k^{\prime }\left|t^{\mathrm{eff}}\right|k\rangle =\frac{\hbar \upsilon_{F}k_{F}}{\pi R^{2}}\int rdr{\int d\phi f(\phi ,\vec{p})}^{\left(1\;se^{-i\theta k^{\prime }}\right)}\left(\begin{array}{c} 1\\ se^{i\theta k} \end{array}\right)e^{-i{\vec{k}}^{\prime }\cdot \vec{r}}\frac{e^{ikr}}{\sqrt{r}}=\;\\ =\hbar \upsilon_{F}k_{F}[1+e^{-i(\theta_{k^{\prime }}-\theta_{k})}]\frac{1}{\pi R^{2}}\int_{0}^{R}rdr\frac{e^{ikr}}{\sqrt{2\pi kr}}{\sum_{m}\left(-1\right)}^{m}[e^{2i\delta_{m}}-1]\\ \times \int_{0}^{2\pi }d\phi \;e^{im\phi }e^{-ipr\;}{}^{\cos[\phi -(\theta_{p}-\theta_{k})]} \end{array}$$ (27)

(both waves are normalized to the square root of the area, *πR*^2^). It depends on the energy, *ϵ_p_*, and on the scattering angle, $\theta_{\vec{k}-{\vec{k}}^{\prime }}$ between the incoming and outgoing wave. The factor $\left[1+e^{-i(\theta_{k^{\prime }}-\theta_{k})}\right]$ provides the cancellation of the backward scattering. The integration over *ϕ* gives a Bessel function, *J_m_*(*kr*), and the integral over *r* can be approximated as:

1πR2eim(θp−θk)∫0Rr dreikr2πkr2πimJm(kr)≈eim(θp−θk)πkReiπ4(−1)m1R∫0Rdr (28)

Finally:

$$\langle k^{\prime }\left|t^{\mathrm{eff}}\right|k\rangle =[1+e^{-i(\theta_{k^{\prime }}-\theta_{k})}]\;e^{i\pi /4}\frac{\hbar \upsilon_{F}}{\pi R}\sum_{m}\left[e^{2i\delta_{m}}-1\right]\;e^{im(\theta_{k^{\prime }}-\theta_{k})}$$ (29)

Now, the sum can be performed. Defining *α* as the non-integer part of the flux, $(\vec{p}-\vec{K})\cdot \vec{b}/2\pi =N+\alpha$, with *α* < 1, we obtain $\left(\Theta \equiv \left(\theta_{k^{\prime }}-\theta_{k}\right)\right)$:

$$\langle p\left|t(\vec{k})\right|k\rangle =\left[1+e^{-i\Theta }\right]e^{i\pi /4}\frac{\hbar \upsilon_{F}}{\pi R}\left[-2\pi \delta (\Theta )(1-\cos\pi \alpha )+\sin\pi \alpha \frac{e^{-i(N+1/2)\Theta }}{\sin\Theta /2}\right]$$ (30)

The prefactor of Equation (26), $\left(1-\hat{k}\cdot {\hat{k}}^{\prime }\right)\equiv 1-\cos\Theta$, makes the first term in Equation (30) disappear, which is likely to be spurious anyway [48]. It also compensates for the divergence at Θ = 0 of the second term. The final result is:

$$\frac{1}{\tau \left({\vec{k}}_{F}\right)}=\sin^{2}\pi \alpha \frac{2\upsilon_{F}}{\pi^{4}k_{F}R^{2}}\int_{0}^{2\pi }d\theta_{k^{\prime }}(1-\cos\Theta )\frac{\left(1+\cos\Theta \right)}{\sin^{2}\Theta /2}\sin^{2}\pi \alpha \frac{8\upsilon_{F}}{\pi^{4}k_{F}R^{2}}$$ (31)

Equation (31) depends on the incoming direction due to the orientation of the Burgers vector contained in *α*.

When multiplying this result by the number of dislocations, *z_d_*, and after averaging over their random distribution, the resistivity of Equation (25) due to the elastic scattering on the edge dislocation turns out to be:

$$\bar{\rho }\left({\vec{k}}_{F}\right)=z_{d}\frac{\hbar }{e^{2}}\frac{16}{\pi^{2}}\frac{\bar{\sin^{2}\pi \alpha }}{{\left(k_{F}R\right)}^{2}}=z_{d}\frac{\hbar }{e^{2}}\frac{16}{\pi^{2}}\frac{\sin^{2}\pi /3}{{\left(k_{F}R\right)}^{2}}$$ (32)

The resistivity is proportional to the density of the defects and inversely proportional to the density of carriers $n\propto k_{F}^{2}$. Lattice relaxation, around the branch point, provided it is not too strong, would contribute to the resistivity with a term that is a higher positive power of *k_F_* [20] and would not change this result qualitatively. This is proven in the Section 3.2.

### 3.2. Cross-Section Due to the Stress at the Origin of the Edge Dislocation

The singularity point, which is the origin of the branch cut, due to the edge dislocation, is also the center of a strain texture induced by the defect. In this section, we show that the contribution of the strain to the total cross-section of Equation (23), due to the stress at the dislocation, is higher order with respect to the one that contributes to the relaxation time, *τ* (*k_F_*), calculated in the previous section. The stress provides an extra term coupling the sublattices, A and B, within the same valley $\hat{V}=\int d^{2}r\;V(r)a^{†}(r)b(r)+h.c.$(here *a* (*r*), *b* (*r*) are fields on the two sublattices) which takes the form [32,50]:

$$\begin{array}{ccc} V(r)=\vec{\sigma }\cdot \vec{A}(r)e^{-i\vec{K}\cdot \vec{b}}; & A_{x}(r)=\frac{3}{4}\beta \kappa (u_{xx}-u_{yy})(r); & A_{y}=\frac{3}{2}\beta \kappa u_{xy}(r) \end{array}$$ (33)

where *βκ* is the stiffness of the lattice. Here, *u_ij_* (*r*) is derived from the displacement field around the dislocation center, $\vec{u}(r)$, which is taken to decay radially as 1/*r* by approximation:

$$u_{ij}(r)=\frac{1}{2}\{\partial_{i}u_{j}+\partial_{j}u_{i}\}(r)$$ (34)

Explicitly, the potential arising from the strain due to the edge dislocation, away from the core of the defect (r > *a*) is, in cylindrical coordinates,

$$V(r)=\frac{\beta \kappa }{r}\left(\begin{array}{cc} 0 & -i\cos\theta e^{2i\theta }\\ i\cos\theta e^{-2i\theta } & 0 \end{array}\right)$$ (35)

and is limited to an area < *πℓ*^2^, where *ℓ* is the mean free path between dislocations. The incoming Dirac electron wave function:

$$\Psi_{\vec{k},s}^{0}(\vec{r})=\frac{1}{\sqrt{2}}\left(\begin{array}{c} 1\\ se^{i\theta k} \end{array}\right)e^{i\vec{k}\cdot \vec{r}}$$ (36)

is scattered by the potential. The Green’s function is required, which solves the Dirac equation inclusive of the A-B flux, *f*, at the origin:

$$[\omega 1-H_{f}]G_{f}(r,r^{\prime };\omega )=i\delta (\vec{r}-{\vec{r}}^{\prime })\sigma_{z}$$ (37)

Its spectral representation is given in Appendix B. In the Born approximation, we get:

$$\Psi_{\vec{k},s}(\vec{r})=\Psi_{\vec{k},s}^{0}(\vec{r})+\sigma_{z}\int d{\vec{r}}^{\prime }G_{f}(\vec{r},{\vec{r}}^{\prime },\omega )V({\vec{r}}^{\prime })\Psi_{\vec{k},s}^{0}({\vec{r}}^{\prime })$$ (38)

We keep just the contribution coming from the pole Equation (A20), and we take the direction of the incoming $\vec{p}$ orthogonal to $\vec{b}$ as the reference direction.

Using the decomposition of a plane wave in angular momentum eigenfunctions, Equation (36) becomes:

$$\Psi_{\vec{k},s}^{0}(r,\theta_{r})=\frac{1}{\sqrt{2}}\sum_{n}i^{n}\left(\begin{array}{c} J_{n}(kr)\\ isJ_{n+1}(kr)e^{i\theta_{r}} \end{array}\right)e^{in(\theta_{r}-\theta_{k})}$$ (39)

The integral giving the scattered part of the wave function is (*B* is a constant, including *βκ* and a normalization factor):

σz∫dr→′Gf(r→,r→ ′,ω)V(r→′)Ψk→ ,s0==σz(−i)fBk(sJm+f(kr)−iJm+1+f(kr)eiθr)∫d r′dθr′∑meim(θr−θr′)×∑n(Jm(kr′)Jn+1(kr′)cosθr′e3iθr′+Jn(kr′)Jm+1(kr′)cosθr′e−3iθr′)einθr′=(−i)fBk∑n{I1n+I2n} (40)

where *I_in_* are shorthand for the two contributions to the integral given above. Let us analyze the first one:

$$\begin{array}{l} I_{1n}=\sum_{m}\left(\begin{array}{c} sJ_{m+f}(kr)\\ iJ_{m+1+f}(kr)e^{i\theta_{r}} \end{array}\right)e^{im\theta_{r}}\int dr^{\prime }d\theta_{r^{\prime }}J_{m}(kr^{\prime })J_{n+1}(kr^{\prime })\cos\theta_{r^{\prime }}e^{3i\theta_{r^{\prime }}}e^{i(n-m)\theta_{r^{\prime }}}\\ =\left(\begin{array}{c} sJ_{n+4+f}(kr)\\ iJ_{n+5+f}(kr)e^{i\theta_{r}} \end{array}\right)e^{i(n+4)\theta_{r}}\int dr^{\prime }J_{n+4}(kr^{\prime })J_{n+1}(kr^{\prime })\\ +\left(\begin{array}{c} sJ_{n+2+f}(kr)\\ iJ_{n+3+f}(kr)e^{i\theta_{r}} \end{array}\right)e^{i(n+2)\theta_{r}}\int dr^{\prime }J_{n+2}(kr^{\prime })J_{n+1}(kr^{\prime }) \end{array}$$ (41)

These integrals are special cases of the Weber–Schafheitlin integral:

$$\int_{0}^{\infty }dtJ_{\alpha +p}(kt)J_{\alpha -p-1}(kt)=\frac{{\left(-1\right)}^{p}}{2k}$$ (42)

with (*α*, *p*) = (*n* + 3, 1) and (*n* + 2, 0). Thus, the first integral is equal to −1/2*k*, while the other is 1/2*k*. Putting things together:

$$I_{1n}=-\frac{1}{2k}\left(\begin{array}{c} sJ_{n+4+f}(kr)\\ iJ_{n+5+f}(kr)e^{i\theta_{r}} \end{array}\right)e^{i(n+4)\theta_{r}}+\frac{1}{2k}\left(\begin{array}{c} sJ_{n+2+f}(kr)\\ iJ_{n+3+f}(kr)e^{i\theta_{r}} \end{array}\right)e^{i(n+2)\theta_{r}}$$ (43)

Similarly, the second contribution yields:

$$\begin{array}{l} I_{2n}=\sum_{m}\left(\begin{array}{c} sJ_{m+f}(kr)\\ iJ_{m+1+f}(kr)e^{i\theta_{r}} \end{array}\right)e^{im\theta_{r}}\int dr^{\prime }d\theta_{r^{\prime }}J_{n}(kr^{\prime })J_{m+1}(kr^{\prime })\cos\theta_{r^{\prime }}e^{-3i\theta_{r^{\prime }}}e^{i(n-m)\theta_{r^{\prime }}}\\ =\left(\begin{array}{c} sJ_{n-4+f}(kr)\\ iJ_{n-3+f}(kr)e^{i\theta_{r}} \end{array}\right)e^{i(n-4)\theta_{r}}\int dr^{\prime }J_{n-4}(kr^{\prime })J_{n+1}(kr^{\prime })\\ +\left(\begin{array}{c} sJ_{n-2+f}(kr)\\ iJ_{n-1+f}(kr)e^{i\theta_{r}} \end{array}\right)e^{i(n-2)\theta_{r}}\int dr^{\prime }J_{n-2}(kr^{\prime })J_{n+1}(kr^{\prime }) \end{array}$$ (44)

where the same integral as Equation (42) appears, with (*α*, *p*) = (*n* − 1, −3) and (*n*, −2), so that the final result is:

σz∫dr→′G(r→,r→′,ω)V(r→′)Ψs,n0(r→′)==(−i)fA2einθ{−(sJn+4+f(kr)sJn+5+f(kr)eiθr)ei4θr+(sJn+2+f(kr)sJn+3+f(kr)eiθr)ei2θr−(sJn−4+f(kr)sJn−3+f(kr)eiθr)e−i4θr+(sJn−2+f(kr)sJn−1+f(kr)eiθr)e−i2θr} (45)

The dominant contributions to the *t* – matrix scattering amplitude in the long wavelength limit $\left(\vec{k}\to 0\right)$ come from the terms in which the order of the Bessel functions is the lowest possible, *i.e*.:

$$\begin{array}{ccc} \langle k^{\prime }\left|t^{\mathrm{eff}}\right|k\rangle \to \langle \Psi_{s,0}^{0}\left|\sigma_{z}GV\Psi_{s,\pm 2}^{0}\right|\rangle & and & \langle \Psi_{s,0}^{0}\left|\sigma_{z}GV\Psi_{s,\pm 4}^{0}\right|\rangle \end{array}$$ (46)

Putting aside the consideration that these matrix elements imply incoming waves of relatively high order (*i.e.*, |*n*| = 2, 4), the largest ones among them lead to integrals whose limiting form is of the kind:

$$\underset{k\to 0}{\lim}\frac{1}{R^{2}}\int_{0}^{\infty }rdrJ_{0}(kr)J_{f}(kr)\approx \frac{1}{k^{2}R^{2}\Gamma (1+1/3)}{\int_{0}^{k\ell }\left(\frac{1}{2}t\right)}^{1/3}J_{0}(t)∼{\left(k\ell \right)}^{1/3}$$ (47)

We conclude that their contribution to the cross-section goes as:

$$\sigma_{\mathrm{disl}}^{\mathrm{stress}}∼\frac{\ell^{2}}{R^{2}}{\left(k_{F}\ell \right)}^{2/3}$$ (48)

which is higher order, when compared with $\sigma_{\mathrm{disl}}^{\mathrm{topological}}∼{\left(k_{F}R\right)}^{-2}$, which was derived in Section 3.1.

### 3.3. Self-Energy of the Dislocation

We now evaluate the formation the self-energy of the dislocation. In the long wavelength limit, we cannot account for the cost of the pentagon-heptagon defect formation, but we can include the strain cost, which is long range, because it decays as 1/*r*. We will make use of the Hellmann–Feynman theorem [51]:

$$\frac{dE(\lambda )}{d\lambda }=\langle \Psi_{0}^{\lambda }\left|\hat{V}\right|\Psi_{0}^{\lambda }\rangle$$ (49)

by adding a perturbation potential, $\lambda \hat{V}$, of the kind of Equation (35) to the unperturbed Hamiltonian $H_{0}=-i\upsilon_{F}\vec{\sigma }\cdot \vec{\nabla }$:

$$\begin{array}{cc} \text{with} & \langle \lambda \hat{V}\rangle \end{array}=\frac{-i}{2}\int d^{2}r\int_{-\infty }^{+\infty }d\omega \;e^{i\omega \eta }\underset{r^{\prime }\to r}{\lim}\text{tr}\left[\omega 1+i\upsilon_{F}\vec{\sigma }\cdot \vec{\nabla }\right]\{G^{\lambda }(r,r^{\prime },\omega )-G^{0}(r,r^{\prime },\omega )\}$$ (50)

*G*^λ^ is the Green function for our system, with an A-B flux, λ*f*, at the origin. λ is a parameter introduced for convenience, which we integrate out from zero to one:

$$E-E_{0}=\int_{0}^{1}\frac{d\lambda }{\lambda }\langle \lambda \hat{\text{V}}\rangle$$ (51)

We have subtracted a reference term in which the dislocation is absent. This is independent of λ and is immaterial, except for the fact that it provides the vanishing of the perturbation, when λ → 0. The prescription, *e^i^^ωη^*, is fixed by the requirement that one has to impose *t′ → t* + 0^+^ to get the correct ordering in the Green’s functions, which is $\langle {\hat{\psi }}^{†}(r^{\prime },t^{\prime })\hat{\psi }(r,t)\rangle$.

Using the Dyson equation $G^{\lambda }=G_{0}^{0}+G_{0}^{0}\lambda V\;G^{\lambda }$, we obtain:

$$E-E_{0}=\frac{-i}{2}\int d^{2}r\int_{0}^{1}\frac{d\lambda }{\lambda }\int_{-\infty }^{+\infty }d\omega \;e^{i\omega \eta }\underset{r^{\prime }\to r}{\lim}\text{tr}\left\{\left[\omega 1+i\upsilon_{F}\vec{\sigma }\cdot \vec{\nabla }\right]\int d^{2}r^{\prime \prime }G_{0}^{0}(r,r^{\prime \prime },\omega )\lambda V(r^{\prime \prime })G^{\lambda }(r^{\prime \prime },r^{\prime },\omega )\right\}$$ (52)

The strain potential of the edge state is taken from Equation (35). It is defined for *r > a*, where *a* is the radius of the core of the dislocation.

According to Equation (A13), $\left[\omega 1+i\upsilon_{F}\vec{\sigma }\cdot \vec{\nabla }\right]G_{0}^{0}(r,r^{\prime \prime },\omega )=i\delta (r-r^{\prime \prime })\sigma^{z}$. On the other hand, the spectral representation of *G*^λ^, Equation (A25), which includes the half pole only $\left(\hbar \upsilon_{F}\equiv 1\right)$, is:

$$G^{\lambda }(\vec{r},{\vec{r}}^{\prime },\omega )\to -i(-i)\lambda f\frac{\omega }{\pi }\sum_{m,s}\frac{1}{2}\left(\begin{array}{cc} J_{m+\lambda f}(pr)J_{m}(pr) & i\;J_{m+\lambda f}(pr)J_{m+1}(pr)e^{-i\theta_{r}}\\ -iJ_{m+1+\lambda f}(pr)J_{m}(pr)e^{-i\theta_{r}} & J_{m+1+\lambda f}(pr)J_{m+1}(pr) \end{array}\right)$$ (53)

The correct time ordering requires that, before taking the limit, the variables are exchanged: *r′* → *r* and *θ_r_* *↔−θ_r′_*. After multiplying the matrices and taking the trace, we get:

$$\begin{array}{l} \int d^{2}r\underset{r^{\prime }\to r}{\lim}\sigma^{z}\lambda V(r)G^{\lambda }(r,r^{\prime },\omega )\approx \\ \frac{-\lambda }{4\pi }\int rdr\frac{\beta k}{r}\int_{0}^{2\pi }d\theta (-i\cos\theta ){\left(i\right)}^{1-\lambda f}\omega \\ \times \sum_{m}\left[e^{2i\theta }e^{-i\theta }J_{m+1+\lambda f}(\omega r)J_{m}(\omega r)+e^{-2i\theta }e^{i\theta }J_{m+\lambda f}(\omega r)J_{m+1}(\omega r)\right]\\ =\frac{-\lambda }{4\pi }\int rdr\;\pi \frac{\beta }{r}(-i){\left(i\right)}^{1-\lambda f}\omega \\ \times \sum_{m}\left[J_{m+1+\lambda f}(\omega r)J_{m}(\omega r)+J_{m+\lambda f}(\omega r)J_{m+1}(\omega r)\right]\\ \text{with}\;\sum_{m}\left[\ldots \right]=[J_{1+\text{λ}f}(2\omega r)+J_{\lambda f-1}(2\omega r)]=\frac{2\lambda f}{\omega r}J_{\lambda f}(2\omega r) \end{array}$$ (54)

Now the integral over *ω*. The largest contribution comes from *ω* *→* 0 and/or *r →* 0. Hence, we substitute the factor, *ω*, with sin(*ωa*)*/a*, to regularize the integral. Using dimensionless variables, *t* = 2*ωr*, *b* = *a/*(2*r*) *→* 0, we perform the integration on the real axis:

$$\begin{array}{l} \frac{\lambda f}{ar}\underset{b\to 0}{\lim}\int_{-\infty }^{\infty }dt\frac{\sin bt}{t}J_{f}(t)=\frac{\lambda f}{ar}\underset{b\to 0}{\lim}\left[\int_{0}^{\infty }dt\frac{\sin bt}{t}J_{\lambda f}(t)+\int_{0}^{\infty }dt\frac{\sin bt}{t}J_{\lambda f}(-t)\right]\\ =\frac{\lambda f}{ar}\underset{b\to 0}{\lim}(1+e^{i\pi \lambda f})\int_{0}^{\infty }dt\frac{\sin bt}{t}J_{\lambda f}(t)=\frac{2\lambda f}{ar}e^{i\pi \lambda f/2}\cos\frac{\pi \lambda f}{2}\times \frac{\cos\pi \lambda f}{\lambda f} \end{array}$$ (55)

Plugging all together, the awkward prefactor, *e^iπλf/^*^2^, disappears, and the final result is (*`* is the size of the defect):

$$E-E_{0}=-\frac{1}{2}\frac{\beta k}{a}\int_{0}^{\ell }\frac{dr}{r}\int_{0}^{1}d\lambda \;\text{cos}\frac{\pi \lambda f}{2}\times \cos\;\pi \lambda f=\frac{1}{2}\frac{\beta k}{a}\int_{0}^{\ell }\frac{dr}{r}\frac{1}{6\pi }=\frac{1}{12\pi }\frac{\beta k}{a}\text{ln}\frac{\ell }{a}$$ (56)

This is the expected self-energy for a long-range strain potential.

## 4. Effective Theory for Boundary States in 3D Topological Insulators

Bismuth-based materials are mostly studied since the prediction that Bi_(1_*_−x_*_)_Sb*_x_* is a strong 3D TI [52], which was confirmed soon after with angle-resolved photoemission spectroscopy (ARPES) [53]. The stoichiometric crystal, Bi_2_Se_3_, is the prototype of a class of 3D TIs. This material was predicted to have boundary states with energy dispersion forming a Dirac cone centered at the Γ point located within the insulating gap [54]. The prediction has been experimentally confirmed [55]. The atomic structure of the material consists of the stacking of quintuple layers. While the coupling is strong inside each of the quintuple layers, the coupling between quintuple layers is much weaker, predominantly of the van der Waals type. The non-trivial topology in the band structure of the material stems from the inversion, at the Γ point, of the bands of the *p_z_* orbital character, arising from Se and Bi atoms, due to the strong spin orbit (SO) coupling. A minimal tight-binding model represents the wavefunction for the valence and conduction bands of opposite parity, on the basis of four *p*− orbitals, spanning the spin and the orbital degree of freedom, Φ*^T^* = (*|p*1*_z_* *↑*〉*, |p*2*_z_* *↑*〉*, |p*1*_z_* *↓*〉*, |p*2*_z_* *↓*〉) (the upper label, *^T^* , means “transpose”) as:

$$|\Psi_{n\vec{k}}(r)\rangle =\sum_{R}e^{i\vec{k}\cdot \vec{R}}\sum_{\alpha =A,B,C,D}\Psi_{n\alpha }(\vec{R})|\Phi_{\alpha }\left(\vec{r}-\vec{R}\right)\rangle$$ (57)

where $\vec{R}$ are lattice vectors, in terms of the slowly-varying envelope functions Ψ*_n_*_α_( $\vec{R}$). The symmetries of the crystal can be represented as:

- the time reversal symmetry $\Theta =i\sigma_{y}⊗IK$
- the inversion symmetry $L=I⊗\tau_{z}$
- the three-fold rotational symmetry around the *z*-axis $C_{3}=\exp(i\frac{\pi }{3}\sigma_{z}⊗I)$

Here, K is the complex conjugation, *σ_i_* are the Pauli matrices in the spin space and *τ_i_* are the Pauli matrices acting in the orbital space.

For states with $\vec{k}$ in the vicinity of the Γ point, the Hamiltonian can be written in the $\vec{k}\cdot \vec{p}$ or effective mass approximation as [56]:

$${Η}_{0}-µ{\text{II}}_{4\times 4}=(h_{0}-µ){\text{II}}_{4\times 4}+\hbar \upsilon_{F}\left(\begin{array}{cccc} -ℳ-µ & i\partial_{z} & 0 & i(\partial_{x}-i\partial_{y})\\ i\partial_{z} & -ℳ-µ & i(\partial_{x}-i\partial_{y}) & 0\\ 0 & i(\partial_{x}+i\partial_{y}) & -ℳ-µ & -i\partial_{z}\\ i(\partial_{x}+i\partial_{y}) & 0 & -i\partial_{z} & ℳ-µ \end{array}\right)$$ (58)

where *v_F_* is the Fermi velocity close to the chemical potential, *μ. h*_0_ *− μ* does not contribute with any interesting feature to the model and will be dropped in the following. In the bulk, *−i* $\vec{\nabla }$ *→* $\vec{k}$and $ℳ\to ℳ(k)=M+C_{1}k_{z}^{2}+C_{2}(k_{x}^{2}+k_{y}^{2})$ with *M >* 0 and *C*_1_*, C*_2_ *<* 0, to implement the inversion of the bulk bands [56]. The relative sign between *M* and *C*_1_*_,_*_2_ qualifies the insulator as being topologically non-trivial or trivial. *ħv_F_* *M* is the bulk gap of the two-band model. The bulk Hamiltonian can be rewritten in compact form as:

$$H_{0}(k)=\hbar \upsilon_{F}[{\hat{\gamma }}^{0}M+{\hat{\gamma }}^{i}k_{i}]$$ (59)

where ${\hat{\gamma }}^{0}{}^{\;}=I_{2\times 2}⊗\tau_{z}$, ${}^{\;}{\hat{\gamma }}^{1}=\sigma_{x}⊗\tau_{x}$, ${}^{\;}{\hat{\gamma }}^{2}=-\sigma_{y}⊗\tau_{x}\;{\hat{\gamma }}^{3}=\sigma_{z}⊗\tau_{x}$. Note that these matrices satisfy the Clifford algebra {γ*^i^*, γ*^j^*} = *η^ij^*, *i.e.*, they are a set of Dirac matrices. This means that they can be rotated as every well-educated set of Dirac matrices.

Other important symmetries that could be present are the particle-hole symmetry, Ξ, and the chiral symmetry, Γ. $\Xi =\sigma_{x}(-i\tau_{y})K$ is an antiunitary symmetry, such that Ξ*^T^* *H*(*k*)Ξ = *−H*(*−k*). The chiral symmetry Γ = *i ·* ΘΞ is a unitary transformation that changes the sign of the Hamiltonian without reversing the sign of $\vec{k}$. In the presence of a non-zero chemical potential, the Hamiltonian of Equation (58) has neither the particle-hole symmetry, Ξ, nor the chiral symmetry, Γ. As Θ^2^ = *−*1, Ξ^2^ = 0, Γ^2^ = 0, it belongs to the class, *AII*, of the Altland–Zirnbauer classification [57].

In Appendix C, we derive the boundary states for a flat surface orthogonal to the *z*^*−*axis, with the simplifying assumption that *C*_1_*_,_*_2_ = 0 and that *M* changes sign at the boundary. This provides localized states at the boundary, which decay exponentially with the same decay length 1*/M* on both sides of the boundary. The simplifying assumption allows for an analytical treatment of the matching condition [29]. The reduced Hamiltonian for the boundary states conserves their helicity, $\vec{\sigma }\cdot \vec{k}$.

## 5. Possibility of Gapless Bound States at a Screw Dislocation in a 3D TI

**Figure 2.**
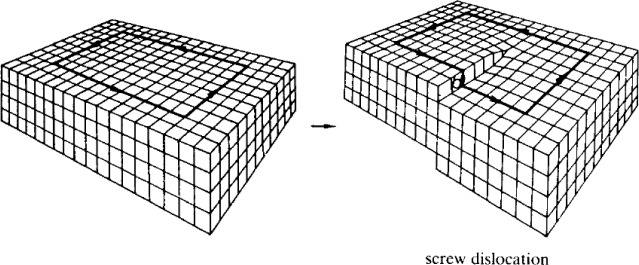
Volterra process for the screw dislocation (taken from [45]). In the picture, the Burgers vector is one lattice spacing long and points downward.

In a 3D material, a screw dislocation can occur. The Volterra process for the creation of such a defect requires cutting the material along a half plane. Next, the two free surfaces are twisted with a relative displacement along the direction of the plane and glued back, so that the right-hand side is displaced upward and the left-hand side is displaced downward, as shown in Figure 2. It has been shown that, in a 3D TI with inversion symmetry, a screw dislocation of Burgers vector, $\vec{b}$, harbors two gapless Dirac modes, which propagate helically along the screw axis, but are localized in the radial direction, provided the constraint:

$$M_{v}\cdot \vec{b}=\pi (\mathrm{mod}2\pi )$$ (60)

is fulfilled [34]. Here, M*_ν_* = (*ν*_1_G_1_ + *ν*_2_G_2_ + *ν*_3_G_3_))*/*2 is a time reversal invariant momentum (TRIM) expressed on the basis of reciprocal lattice vectors (G_1_*,* G_2_*,* G_3_). (*ν*_1_*, ν*_2_*, ν*_3_) are the “weak topological invariants”, which contribute in classifying the 3D TI [52]. One starts by deriving the *Z*_2_ variables *δ*(Γ*_i_*) = *±*1 from the parities of the occupied bands. Here, Γ*_i_* are the time reversal invariant *k−*vector points in the Brillouin zone. By choosing appropriate gauges for the Bloch functions, it is possible to obtain that a single one, *δ*(M*_ν_*), takes the opposite sign with respect to the others. In the case of Bi_2_Se_3_ discussed above, there is no such non-vanishing M*_ν_* point, and M*_ν_* is set equal to the Γ *≡* (0*,* 0*,* 0) point. Hence, the constraint of Equation (60) cannot be fulfilled. As Bi is very close to a band inversion transition at the *L* point, a good test material that can satisfy the constraint of Equation (60) is the strong TI, Bi_1_*_−x_*Sb*_x_* (with *x* *∼* 0.03), of topological invariants *ν*_0_ = 1 and (*ν*_1_*, ν*_2_*, ν*_3_) = (1*,* 1*,* 1) [58]. If *~b* is one lattice spacing along one of the three directions of G*_i_*, let us say *x*^_3_, the condition is fulfilled.

In the case of the screw dislocation, the Burgers vector is oriented along the defect line, at difference with the edge dislocation, which has the Burgers vector orthogonal to the defect axis. The change of coordinates, describing a screw dislocation with $\vec{b}=(0,0,-b)$, is:

$$\begin{array}{l} x^{\bar{1}}=x^{1}\\ x^{\bar{2}}=x^{2}\\ x^{\bar{3}}=x^{3}-\frac{b}{2\pi }\arctan\frac{x^{2}}{x^{1}} \end{array}$$ (61)

The coordinates with overlined indexes refer to the local inertial set of coordinates, *i.e.*, the $x^{\bar{a}}$ be interpreted as the coordinates before the Volterra process takes place. A former $x^{\bar{3}}=const$ plane existing before the creation of the defect, changes when the defect is created in such a way that the *x*^3^ coordinate is displaced by *b*, when circulating of an angle *θ* = 2*π* around the vertical axis. The corresponding tetrads are:

$$e^{a}{}_{µ}=\left(\begin{array}{ccc} 1 & 0 & 0\\ 0 & 1 & 0\\ \frac{b}{2\pi }\frac{x_{2}}{x_{1}^{2}+x_{2}^{2}} & -\frac{b}{2\pi }\frac{x_{1}}{x_{1}^{2}+x_{2}^{2}} & 1 \end{array}\right)$$ (62)

while the inverse tetrads are:

eaµ=(100010−b2πx2x12+x2 2b2πx1x12+x221) (63)

The only non-vanishing component of the torsion is $T_{12}{}^{\bar{3}}=-b\delta (\vec{r})$, while the curvature vanishes, as in the case of the edge dislocation. The rotation of the Dirac matrices gives (with *ħυ_F_* = 1):

$${ℋ}_{screw}(\vec{k})={ℋ}_{M_{v}}(\vec{k})+i\frac{1}{2\pi r}\vec{\gamma }\cdot \hat{\theta }\vec{b}\cdot \vec{\nabla }$$ (64)

Here, ${{ℋ}_{M}}_{\nu }$ is the linearized $\vec{k}\cdot \vec{p}$ Hamiltonian in the vicinity of the M*_ν_* point. In the case of Bi_1_*_−x_*Sb*_x_*, the Time Reversal Invariant Momentum (TRIM) is one of the three equivalent LTRIMs (which is (1*,* 1*,* 1) in the appropriate G vector basis), where the band inversion occurs. The ${{ℋ}_{M}}_{\nu }(\vec{k})$ in the $\vec{k}\cdot \vec{p}$ approximation, to first order in $\vec{k}$, has been worked out in [58] and is unitarily equivalent to the Hamiltonian of Equations (58) and (59) [59]. Strictly speaking, when choosing an approximated wave function of the type $e^{iM\upsilon \cdot \vec{r}}\Psi (\vec{r})$, where:

$$\Psi \left(r,\theta ,x^{\bar{3}}\right)=\Phi (r,\theta )e^{ik_{\bar{3}}x^{\bar{3}}}$$ (65)

is the slowly varying part in cylindrical coordinates, the coordinates $\vec{r}=(r,\theta )$ are in the flat reference frame, as well as the polar axis (*i.e.*, overlined variables $x^{\bar{i}}$). The Schrödinger equation for Φ(*r, θ*) is the Dirac equation with an Aharonov–Bohm flux given by $2\pi \Omega =M_{\nu }\cdot \vec{b}$ and eigenvalue $E=E(k_{\bar{3}})$:

$$\left\{-i\left[\vec{\gamma }\cdot \hat{r}\partial_{r}+\frac{\vec{\gamma }\cdot \hat{\theta }}{r}(\partial_{\theta }-i\Omega )\right]+\vec{\gamma }\cdot \hat{z}k_{\bar{3}}+M\gamma 0\right\}\Phi (r,\theta )=E\;\Phi (r,\theta )$$ (66)

In case $M_{v}\cdot \vec{b}=\pm \pi$, two helical states with gapless linear energy dispersion are bound to the screw dislocation. Indeed, a zero energy solution of Equation (66) can be found, exponentially decaying away from the dislocation core, with the antiperiodic boundary condition:

Φ(r,θ)=−Φ(r,θ+2π) (67)

It follows that the full wavefunction $e^{iM_{v}\cdot \vec{r}}\Psi (\vec{r})$, when expressing $x^{\bar{3}}$ in terms of *x*^3^, acquires an extra phase $e^{iM_{v}\cdot \vec{b}}=-1$, which compensates the antiperiodic boundary condition given above, due to the fact that *x*^3^ has to be displaced by *b*.

We explicitly derive Ψ. According to Equation (66) $\left(k_{\bar{3}}\to k\right)$, the explicit equations to be solved are:

$$\begin{array}{l} \left(E-M)\Psi_{A}(\vec{\text{r}})=i\frac{\partial }{\partial z}\Psi_{B}(\vec{\text{r}})+i\;e^{i\theta }\left[\frac{\partial }{\partial r}+\frac{i}{r}\frac{\partial }{\partial \theta }+\frac{1}{2r}\right]\Psi_{D}(\vec{\text{r}}\right)\\ \left(E+M)\Psi_{B}(\vec{\text{r}})=i\frac{\partial }{\partial z}\Psi_{A}(\vec{\text{r}})+i\;e^{i\theta }\left[\frac{\partial }{\partial r}+\frac{i}{r}\frac{\partial }{\partial \theta }+\frac{1}{2r}\right]\Psi_{C}(\vec{\text{r}}\right)\\ \left(E-M)\Psi_{C}(\vec{\text{r}})=i\;e^{-i\theta }\left[\frac{\partial }{\partial r}-\frac{i}{r}\frac{\partial }{\partial \theta }-\frac{1}{2r}\right]\Psi_{B}(\vec{\text{r}})-i\frac{\partial }{\partial z}\Psi_{D}(\vec{\text{r}}\right)\\ \left(E+M)\Psi_{D}(\vec{\text{r}})=i\;e^{-i\theta }\left[\frac{\partial }{\partial r}-\frac{i}{r}\frac{\partial }{\partial \theta }-\frac{1}{2r}\right]\Psi_{A}(\vec{\text{r}})-i\frac{\partial }{\partial z}\Psi_{C}(\vec{\text{r}}\right) \end{array}$$ (68)

The slowly varying function, Ψ, turns out not to be an eigenstate of the integer angular momentum, *mħ*. A solution of the system given above is:

$$\Psi_{n}\propto \left(\begin{array}{c} \left({Ε}_{n}+Μ){Κ}_{n-1/2}(\kappa r\right)\\ \kappa \;{Κ}_{n-1/2}(\kappa r)\\ 0\\ -i\kappa \;{Κ}_{n-1/2}(\kappa r)e^{-\iota \theta } \end{array}\right)e^{-i(n-1/2)\theta }e^{-ikx^{3}}$$ (69)

The functions, *K_ν_*(*κr*), are the modified Bessel functions decaying at infinity with a length-scale *κ^−^*^1^ to be determined. The only normalizable solution is with *n* = 0, so that we have:

$$\Psi_{0,k,b}\propto \left(\begin{array}{c} \left(E_{0}+M\right)\\ k\\ 0\\ -iMe^{-i\theta } \end{array}\right)K_{-1/2}(kr)e^{i\theta /2}e^{-ikx^{3}}$$ (70)

with the eigenvalue: $E_{0}^{2}-M^{2}=k^{2}-k^{2}.$By posing *κ*^2^ = *M*^2^, the energy dispersion is gapless and linear: $E_{0}^{2}=k^{2}.$The solution satisfies antiperiodic boundary conditions in the *θ* variable, as required. Note that the same result is obtained for *ℳ* (*k*) = (*M* + *C*_1_*k*^2^), provided we choose *κ* = *|M* + *C*_1_*k*^2^*|*.

The state, which is time reversed with respect to Equation (70), is:

$$\Psi_{0,-k;-b}\propto \left(\begin{array}{c} 0\\ iMe^{i\theta }\\ -(E_{0}+M)\\ -k \end{array}\right)K_{1/2}(kr)e^{-i\theta /2}e^{ikx^{3}}$$ (71)

These states have opposite helicity. At zero energy, *i.e.*, with *k* = 0, Θ commutes with the Hamiltonian, and one can construct eigenstates of zero energy from Equations (70) and (71), which are mutually time reversed. This can be easily done by summing and subtracting Equations (70) and (71) together, in the *k →* 0 limit:

$$\begin{array}{l} \Psi_{0,p}(r,\theta )\propto {\left[e^{i\theta /2},ie^{i\theta /2},-e^{-i\theta /2},-ie^{-i\theta /2}\right]}^{T}K_{1/2}(kr)\\ \Psi_{0,p}(r,\theta )\propto {\left[e^{i\theta /2},-ie^{i\theta /2},-e^{-i\theta /2},-ie^{-i\theta /2}\right]}^{T}K_{1/2}(kr) \end{array}$$ (72)

where:

$$\Theta \Psi_{0,p}=\Psi_{0,m}$$ (73)

In the class, *AII*, of the Altland–Zirnbauer [57] classification, the linear defect in 3D has a corresponding topological invariant, *Z*_2_ [60]. The protected gapless modes, which are delocalized along the $\hat{z}$*−*axis, can be combined in chiral form, because the *left-chiral* projector, Π*_L_* = (1 *−* Γ)*/*2, and the *right-chiral* projector, Π*_R_* = (1 + Γ)*/*2, commute with Θ.

## 6. Superconductive Proximity in a 3D TI and Bound States at an Axial Vortex

A superconductor in close contact with a normal metal induces Cooper pairing in it by proximity. The bulk states located at the Fermi energy in the metal penetrate the superconductor, even when their energy is below the energy of the superconducting gap, thanks to the Andreev reflection mechanism. The matching at the boundary builds up a superposition of particles and holes in the metal, which are quasiparticles nicknamed “bogoliubons”. A pairing order parameter is induced in the normal metal within a distance from the boundary, which depends on whether transport in the metal is diffusive or “clean” [61]. Proximity does not necessarily imply the opening of a gap in the metal, in particular when the transparency of the boundary is high.

An undoped 3D TI, being a semiconductor, has a Fermi energy located inside the gap separating the bulk bands. Therefore, in principle, bulk quasiparticle states cannot be involved in the proximity. However, the interface of a TI hosts boundary states, whose energy dispersion is the Dirac cone occupying energies within the band gap. The boundary acts as a semimetal of reduced dimension and proximity can take place. However, the properties of the bogoliubons differ from those of the topologically trivial metal, because orbital and spin degrees of freedom are strongly coupled in the Dirac boundary states.

In the presence of a magnetic field, a vortex can be trapped, piercing the heterostructure in which a 3D TI slab is sandwiched between two conventional superconductors. We assume that the *S/T I/S* heterostructure, forming a slab laying in the *x − y* plane, with flat boundary surfaces at *z* = 0*, L*, is immersed in a magnetic field parallel to the *z−*direction. The interest for this configuration has been recently growing since the work by Fu and Kane [35], which predicts the possibility of binding a Majorana zero energy quasiparticle state at the vortex in this geometry. This could be the elementary brick for building a completely new architecture for quantum information [62–64]. It is interesting that, while a vortex can be viewed as an axial defect, it is not constrained by features of the lattice symmetry, due to its size. On the other hand, it adds in all cases an orbital angular momentum of *π* to the bogolubon. This implies that zero energy quasiparticles bound to the vortex core automatically satisfy the required periodic boundary conditions in the azimuthal angle, and the constraint of Equation (60) does not apply. In this section, we report on results obtained about zero energy bound quasiparticle states that can form in the core of the vortex at the boundary with the superconductors, both in the case of even and odd parity proximity [41]. It is unclear whether the pairing induced in the TI is to be expected to have even or odd parity. It has been proposed that, when doped with few percent of Cu, the Bi_2_Se_3_ becomes a topological superconductor, undergoing the superconducting phase transition with an odd-parity order parameter [65,66]. For this reason, we consider both possibilities, and we have found very different results in the two cases.

Usually, proximity is described in the Nambu basis, of the kind: ${\left[(\psi_{↑},\psi_{↓}),(\psi_{↓}^{†},-\psi_{↑}^{†})\right]}^{T}$; and theHamiltonian takes the compact Bogoliubov de Gennes (BdG) mean field form:

$$H_{BdG}(\vec{k})=\left(\begin{array}{cc} {\hat{h}}_{o} & \hat{\Delta }\\ {\hat{\Delta }}^{†} & -{\hat{h}}^{*}{}_{o} \end{array}\right)$$ (74)

Superconductive proximity induced by an even parity singlet pairing requires that . ${\hat{\Delta }}_{S}^{T}(\vec{k})={\hat{\Delta }}_{s}(-\vec{k}).$The odd-parity triplet pairing satisfies ${\hat{\Delta }}_{p}^{T}(\vec{k})=-{\hat{\Delta }}_{p}(-\vec{k}).$ In the case of the two-band 3D TI, an 8 *×* 8 basis is required for the Hamiltonian of Equation (58), with two types of orbitals, labeled generically with *g/u*, even/odd by inversion, respectively. The Pauli matrices, *σ^a^* and *s^a^*, span the orbital and spin space, respectively, while the Pauli matrices, *τ^a^*, address the particle-hole sectors. In the even parity, s-wave, singlet case, we define ∆*_s_* = 〈*ψ_u↑_ψ_u↓_*〉 = 〈*ψ_g↑_ψ_g↓_*〉 = *−*〈*ψ_u↓_ψ_u↑_*〉 = *−*〈*ψ_g↓_ψ_g↑_*〉, assumed to be independent of the orbital type. This gives rise to a pairing mean field Hamiltonian:

$$H_{\mathrm{pair}}^{s}=-i(\Delta_{s}s_{y}\tau_{+}+h.c.)$$ (75)

In the odd parity case, the pairing is chosen with zero spin projection along the spin quantization axis, which is pinned to the *z−*axis, normal to the surface of the slab (polar ordering). This choice is motivated by the expectation that the superconductive gap can vanish at the interface (*z* = 0*, L*) where the Dirac states are located, if transparency is high. The polar pairing is described by:

$$H_{\mathrm{pair}}^{s}=(\Delta_{p}\sigma_{y}s_{y}\tau_{+}+h.c.)$$ (76)

where ∆*_p_* = 〈*ψ_u↑_ψ_g↓_*〉 = *−*〈*ψ_g↑_ψ_u↓_*〉 = *−*〈*ψ_g↓_ψ_u↑_*〉 = 〈*ψ_u↓_ψ_g↑_*〉 is the odd-parity order parameter. The change in signs in the expectation values for ∆*_p_* arises from the fact that the operators act on a triplet pair with zero spin projection along *z*. The choice of zero spin projection allows for a closer comparison with the s-wave case.

We will choose different representation bases for the two symmetries. They are connected to the Nambu basis by unitary transformations, but differ from it. This is convenient, as it can be shown that the induced even and odd-parity proximities give rise to the same matrix form of the model Hamiltonian, when the two different bases are adopted, each for the two different cases. We consider a vortex line of charge *q* = *±*1 piercing the TI, with its axis orthogonal to the boundary surface, and we use cylindrical coordinates oriented along the *z−*axis.The Hamiltonian is (*ħ_vF_* = 1):

$$H=\left(\begin{array}{cc} H_{+} & s^{z}\sigma^{z}i\partial_{z}\\ s^{z}\sigma^{z}i\partial_{z} & H_{-} \end{array}\right)$$ (77)

where, outside the vortex core, the Hamiltonian blocks, *H_±_*, read:

$$H_{\pm }(r>\xi_{o})=\left(\begin{array}{cccc} \mp ℳ-µ & ie^{-i\theta }(\partial_{r}-\frac{i}{r}\partial \theta -\frac{q}{2r}) & \pm \Delta e^{-iq\theta } & 0\\ ie^{i\theta }(\partial_{r}+\frac{i}{r}\partial \theta +\frac{q}{2r}) & \pm ℳ-µ & 0 & \pm \Delta e^{-iq\theta }\\ \pm \Delta *e^{iq\theta } & 0 & \pm ℳ+µ & -ie^{-i\theta }(\partial_{r}-\frac{i}{r}\partial \theta +\frac{q}{2r})\\ 0 & \pm \Delta *e^{iq\theta } & -ie^{i\theta }(\partial_{r}+\frac{i}{r}\partial \theta -\frac{q}{2r}) & \mp ℳ+µ \end{array}\right)$$ (78)

with:

$$ℳ=\left\{M+C_{2}\left(\partial_{r}^{2}+\frac{1}{2r}\partial_{r}-\frac{1}{4r^{2}}\right)+C_{1}\partial_{z}^{2}\right\}$$ (79)

(*M >* 0 and *C*_1_*, C*_2_ *<* 0). We have added the vector potential associated to the vortex, which, far away from the vortex core, takes the form of a pure singular gauge:

$$\begin{array}{ccc} A_{r}=0, & A_{\theta }(r)=-\frac{1}{r}\partial_{\theta }\chi ; & \chi =q\phi \frac{\theta }{2\pi } \end{array}$$ (80)

(*ϕ* = *hc/*2*e* is the flux unit). The phase factor, *e^iqθ^*, breaks the TR invariance, which holds when ∆*_s_* is real (∆*_p_* is purely imaginary).

Equation (77) is appropriate for the even-parity superconducting correlations, when the basis is:

$$B_{s}\equiv {\left[\psi_{g↑},\psi_{u↓},\psi_{g↓}^{†},-\psi_{u↑}^{†}|\psi_{u↑},\psi_{g↓},\psi_{u↓}^{†},-\psi_{g↑}^{†}\right]}^{T}$$ (81)

while a unitary transformation, which changes the basis to:

$$B_{p}\equiv {\left[\psi_{g↑},\psi_{u↓},\psi_{u↓}{}^{†},-\psi_{g↑}{}^{†}|\psi_{u↑},\psi_{g↓},\psi_{g↓}{}^{†},-\psi_{u↑}{}^{†}\right]}^{T}$$ (82)

transforms the model to describe the odd-parity pairing, provided ∆*_s_* is replaced with ∆*_p_*.

We now search for zero energy excitations corresponding to quasiparticles bound to the vortex. When proximity induces s-wave , singlet superconductive correlations, Majorana quasiparticles are bound to the vortex. The wavefunction decays exponentially, as exp(*−|M||z|*), at the boundary, so that two bound states are localized at the interface with each of the two topologically trivial superconductors. The zero energy eigenstates are found by matching solutions inside and outside the vortex core. Its boundary is defined as a circle of radius *ξ_o_* *∼* *ħv_F_* */*∆. An analytic derivation of the quantum field in the two-band model can be given far away from the vortex core in the limit of *μ* = 0 (mid-gap Majorana bound states (MBS)) [41]. The two zero energy real fermion fields, localized far apart at the two boundary surfaces of the slab, *z* *∼* 0^+^*, L^−^*, in the inside of the TI, outside the vortex core (*r* > *ξ_o_*), take the form:

$$\gamma \left(z∼0^{+}\right)\propto e^{-M\text{​}z}K_{1/2}\left(\Delta \text{​​}\;r\right)\cdot \left\{\left[e^{-i\left(1-q\right)\theta /2}\psi_{g↑}+e^{i\left(1-q\right)\theta /2}\text{​}\psi_{g↑}{}^{†}\right]+i\left[e^{i\left(1-q\right)\theta /2}\psi_{u↑}-e^{-i\left(1-q\right)\theta /2}\psi_{u↑}{}^{†}\right]\right\}$$ (83)

$$\gamma \left(z∼L^{-}\right)\propto e^{-M\left(L-z\right)}K_{1/2}\left(\Delta \;r\right)\cdot \left\{-i\left[e^{i\left(1+q\right)\theta /2}\psi_{g↓}-e^{-i\left(1+q\right)\theta /2}\psi_{g↓}{}^{†}\right]+\left[e^{-i\left(1+q\right)\theta /2}\psi_{u↓}+e^{i\left(1+q\right)\theta /2}\psi_{u↓}{}^{†}\right]\right\}$$ (84)

with *λ* *∼* *|M|*. Here, $K_{\pm 1/2}\left(wr\right)=e^{-wr}/\sqrt{wr}$ are the modified Bessel functions, so that the excitations are localized in the surface plane. The decay length scale is *w^−^*^1^ *∼* *ξ_o_*. Inside the vortex core, the normalizable solution requires an *r−*dependent order parameter, ∆*_s_*(*r*), together with the corresponding vector potential, *A*(*r*), and should be matched with the one given previously at *r* = *ξ_o_*.

Let us compare the two Majorana excitations of Equations (83) and (84). They mix both *u* and *g* orbitals and involve opposite spin orientations. To understand the result, we note that, in the absence of superconductive proximity, the *p* and *h* surface states at the two opposite boundaries, *z* = 0*, L*, have exchanged chiralities. In the spinless case, this can be seen also in a simple, reduced model in which chirality is the *σ_x_* operator:

$$\gamma \left(\underline{r}>\xi_{o},z\right)\propto e^{i\sigma_{z}\varphi \left(\underline{r}\right)/2}e^{-i\sigma_{z}\theta /2}e^{-\frac{r}{\xi_{o}}}e^{\frac{\sigma_{x}}{\hbar \upsilon {\;}_{F}}\int^{z}\tilde{\mu }\left(z^{\prime }\right)dz^{\prime }}\left[{}_{\pm 1}^{1}\right]\;\psi$$ (85)

where *ϕ* = *θ* is the phase of the order parameter and *ψ* is a real spinless fermion. The requirement that the wavefunction decays both radially and inside the TI fixes the eigenvector of *σ_x_*, with eigenvalue ±1, and the chirality of the state follows. At the two surfaces, the z convergency requires opposite eigenvalues.

Now, let us assume the vortex charge to be *q* = 1. The Majorana of Equation (83), *γ*(*z* *∼* 0), does not have any orbital angular momentum in the *z−*direction. It includes spin *↑*, so that the total angular momentum *m_J_* = 1*/*2. The Majorana of Equation (84), *γ*(*z* *∼* *L*), has one unit of orbital angular momentum in the *z−*direction (because its *p/h* components have opposite chirality with respect to those of *γ*(*z* *∼* 0) and its orbital momentum has to fit with the vorticity *q* = 1). It includes spin *↓*, so that the total angular momentum is again *m_J_* = 1*/*2. It looks as if the vorticity of the vortex has been split between the two MBSs having *m_J_* = 1*/*2 each. This reminds one of the half quantum vortex of Sr_2_RuO_4_ [68–70], as just one spin component acquires an orbital angular momentum. This is remarkable as, notwithstanding the fact that the vorticity adds just orbital angular momentum, MBSs are formed in a spin-dependent combination. On the other hand, in the presence of strong SO coupling, just the total angular momentum projection along $\hat{z}$ matters. This result is consistent with the so-called “thick flux limit” of [71], when the magnetic field penetrates extensively into the bulk below the surface, so that the bulk insulating gap is entirely restored. In that paper, however, the correspondence between the opposite chiralities at the two surfaces is not mentioned.

States localized at a vortex core occur also when Cooper pairing induced by proximity in the Hamiltonian of Equation (77) is odd-parity . The change of basis to the one of Equation (82) changes the nature of the bound states trapped at the vortex singularity. They are not of the kind of the ones given in Equations (83) and (84), because they are not MBSs. Instead, they are zero energy surface Andreev bound states (SABS), decaying in an oscillatory fashion within the TI slab (*z >* 0) [72]. The state involving just *u* orbitals takes the form:

$$\begin{array}{l} \Psi_{L}\left(r>{\tilde{\xi }}_{o},\theta ,z>0\right)\propto e^{-ikz}H_{\frac{1}{2}}^{\left(2\right)}\left(iwr\right)\cdot \left\{\left[e^{i\pi /4}e^{i\left(1+q\right)\theta /2}\psi_{u↓}+e^{-i\pi /4}e^{-i\left(1+q\right)\theta /2}\psi_{u↓}^{†}\right]+i\left[e^{-i\pi /4}e^{-i\left(1-q\right)\theta /2}\psi_{u↑}+e^{i\pi /4}e^{i\left(1-q\right)\theta /2}\psi_{u↑}^{†}\right]\right\},\\ \Psi_{L}\left(r<{\tilde{\xi }}_{o},\theta ,z>0\right)∼e^{-k^{\prime }z}F\left(w^{\prime }r\right)\left\{\left[e^{i\left(1+q\right)\theta /2}\psi_{u↓}-e^{-i\left(1+q\right)\theta /2}\psi_{u↓}^{†}\right]+\left[e^{-i\left(1-q\right)\theta /2}\psi_{u↑}+e^{i\left(1-q\right)\theta /2}\psi_{u↑}^{†}\right]\right\} \end{array}$$ (86)

Outside the vortex core $\left(r>{\tilde{\xi }}_{o}\right)$, both *κ* and *w* are complex, so that the function decaying in *z* has also an exponentially decaying factor $\left(\left|k\right|∼\sqrt{M/\left|C_{2}\right|}\right)$. The function of *r* is a Hankel function, $H_{1/2}^{\left(2\right)}\left(i\;wr\right)$, of complex argument, with an oscillating factor, as well as a decaying exponential factor $ℑm\left\{w\right\}∼C_{1}\left|\Delta_{p}\right|/C_{2}$, where we have chosen the gauge in which ∆*_p_* is purely imaginary).

Inside the vortex core $\left(r<{\tilde{\xi }}_{o}\right)$, the amplitude $F(w^{\prime }r)∼H_{1/2}^{\left(1\right)}(w^{\prime }r)+H_{1/2}^{\left(2\right)}(w^{\prime }r)$ is a combination of Hankel functions that converges to zero at the origin (*i.e.*, the point where the order parameter vanishes). In a “hard core” approximation, which does not account for the *r*-dependence of ∆*_p_* and $\vec{A}$ inside the core, the value of $w^{\prime }$ is fixed by matching the two solutions of Equation (86) at the core boundary, ${\tilde{\xi }}_{o}$. Inside the TI slab, the bound state decays exponentially along the vortex axis with a wavevector, $k^{\prime }\approx \left|k\right|$. These behaviors qualify the result as a SABS, which, by inspection, is not an MBS, nor an eigenstate of Time Reversal (TR) and decays along the vortex line, with oscillations depending on *|*∆*_p_|*. It is easy to check that the combinations given here in the asymptotic region out of the vortex core are *L*-chiral, *i.e.*, they form the combinations: $\propto \left[\psi_{u↓}e^{i\theta /2}+i\psi_{u↑}^{†}e^{-i\theta /2}\right]$ and $\propto \left[\psi_{u↑}e^{i\theta /2}-i\psi_{u↓}^{†}e^{-i\theta /2}\right]$.

The partner state to the one given in Equation (86) involves the *ψ_gσ_* field operators, in i the *R—*chiral mate combination, *i.e.*: $\propto \left[\psi_{u↑}e^{i\theta /2}+i\psi_{u↓}^{†}e^{-i\theta /2}\right]$, $\propto \left[\psi_{u↓}e^{i\theta /2}-i\psi_{u↑}^{†}e^{-i\theta /2}\right]$. These excitations involve both spin components.

Away from the mid-gap (*μ* ≠ 0), fermionic excitations can be found, which correspond to circular waves propagating at the interface inward or outward from the vortex singularity and merging into the film by traveling across the slab, along the vortex line, with a radial localization length $\hbar \upsilon F/|\Delta_{p}|$. These states involve both *u* and *g* orbitals and have wavevector $k\sim \sqrt{\mu /|C_{1}|}$[41].

There is no possibility for an MBS to exist, when proximity-induced pairing is odd-parity. The reason can be found in the effective parity of the pairing, which is developed in the TI by proximity. It was shown by Fu and Kane [35] that an even-parity superconductor inducing proximity in a TI acts on Dirac fermions as an effective odd-parity pairing, giving rise to the MBS. Vice versa, an odd-parity proximization can be expected to develop into an effective even-parity pairing, which does not give rise to MBSs.

## 7. Summary and Final Remarks

The bulk of 3D TI exhibits an odd number of time reversal invariant wavevectors in the Brillouin zone, in the vicinity of which the Hamiltonian can be expanded in the $\vec{k}\cdot \vec{p}$ approximation up to linear terms only. Projection of these 3D Dirac points onto the appropriate surface gives rise to a 2D Dirac cone dispersion for boundary states, which conserve helicity $\vec{\sigma }\cdot \vec{k}$ thanks to spin-orbit coupling and are robust with respect to static non-magnetic disorder [73–76]. These properties make boundary states of 3D TI very attractive for applications in spintronics [77]. Conductance of graphene, which is a “weak” 2D TI, due to the presence of the two valleys centered at the two time reversal invariant points, *K* and *K^′^*, has been widely analyzed in these years, including weak localization corrections [78]. Topological defects are also being studied in graphene [32,79–82].

In this work, we have shown that an approach similar to the one often used in graphene [83] can be applied to discuss conduction at the boundaries of topological insulators. In the long wavelength limit for Dirac electrons, it is possible to consider a topological defect as a metric distortion in curved space, so that a rotation of the Gamma matrices and a spin connection inherited by the curvature modifies the scattering properties of the carriers [45,84]. Far away from the defect, these modifications can be accounted for by gauge fields. Short-range potentials due to local stress in the lattice can be included in the Born approximation to the Dyson scattering equation. The t-matrix for the scattering of 2D Dirac electrons and edge dislocation has been derived in Section 3.1.

To make a connection with reality, we have interpreted the results as the modeling of one single edge dislocation in graphene for electrons belonging to one valley only, with no inter-valley scattering. In a picture in which defects are considered as non-interacting and very dilute, a Boltzmann approach to conductivity is acceptable, when averaging over their orientation. The contribution to the resistivity is found to be, of course, proportional to the density of defects, but inversely proportional to the density of carriers, in agreement with the measured resistivity. We have also derived the self-energy of the defect connected with the stress involved in the rearrangement of the lattice in Section 3.3. This information could be relevant to estimate the temperature at which the Boltzmann approach breaks down and a defect-mediated phase transition to a disordered phase takes place. As we find a log-dependence of the self-energy on the size of the dislocation, one could surmise that the transition is of Kosterlitz–Thouless type, as in 2D crystal melting. In this case, a temperature scale is determined the stiffness of the lattice, *βκ*. Assuming $\beta ku_{ij}\sim 10\hbar \upsilon_{F}{}^{-1}$, where *u_ij_* is the strain tensor and *v_F_* is the Fermi velocity [32], the energy of the formation of such a defect would turn out to be of the order of tens of electron volts, leading to various thousand of Kelvin [85]. However, the gauge theory cannot exclude cubic terms, which could drive the phase transition to first order [86,87].

On the same lines, the corresponding topological defects in a 3D TI are the screw dislocations. The odd Dirac point, which is responsible for the material being topologically non-trivial, is the best candidate for hosting the branch point of a screw dislocation. Emphasis is put in our work on the fact that the defect could harbor gapless helical electron states propagating along the dislocation axis. Again, we approach the description of boundary states at a flat surface of a 3D TI and at the defect in the long wavelength limit. We have shown that an analytic form of the bound state wavefunctions can be given easily, because the dislocation acts as an effective flux in the 3D Dirac Hamiltonian. The gapless states exist, provided the constraint on the Burgers vector of Equation (60) is satisfied [34]. TIs, like Bi_2_Se_3_ and Bi_2_Te_3_, which have the 3D Dirac point at Γ, cannot fulfill the constraint, while the alloy, Bi_1_*_−x_*Sb*_x_*, could. The constraint on the orientation of the screw dislocation would influence a statistical approach to the proliferation of defects and thoroughly change the conduction properties, as well as the crystal melting. To our knowledge, this topic has not been addressed yet in the literature.

When a 3D TI is sandwiched between two even-parity superconductors, a vortex piercing the structure can host a zero energy bound state, which is a real fermion field. This is a Majorana bound state. There is great excitement at present for the possibility of revealing Majorana bound states in Josephson junctions between TI, in proximity with superconductors [88] and at vortices [66,89]. In this case, the constraint of Equation (60) does not apply, and the reference to the TRIM, which originates the surface Dirac cone, is unnecessary.

The conserved quantity is total angular momentum along the vortex axis due to SO. The two Majorana at opposite surfaces have a total angular momentum of 1/2 along the vortex axis, and the sum matches the unitary vortex orbital momentum (the “vortex charge”). However, it is remarkable that, because of their opposite chirality, one of them has no orbital angular momentum and spin polarization up, while the other one has spin angular momentum down, to be subtracted from one positive unity of orbital angular momentum. Hence, opposite chiralities imply that the orbital angular momentum of the vortex fixes the spin content of the Majorana fields. It is also interesting that, in the case the induced superconductivity by proximity is of the odd-parity type, the zero energy states are Andreev bound states localized at the free surface, with damped oscillations away from the surface.

An alternative route to realize in a controlled way structures with emerging MBS excitations could be to resort to pertinently designed Josephson junction rings [88] and networks. It has been shown that, by properly designing the network, it is possible to realize topological configurations [90–92], which are expected to enucleate defects hosting MBSs [93,94], similar to the ones predicted at the interfaces between a TI and a superconductor.

## Figures and Tables

**Figure A1.**
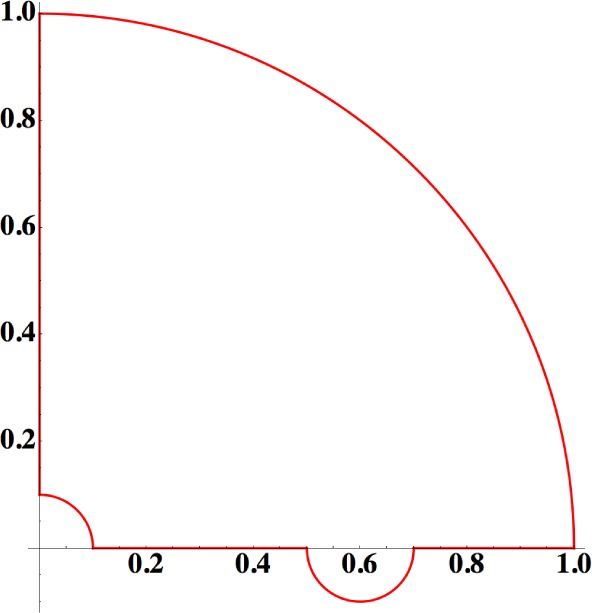
Integration path in the case of an edge dislocation.

## Appendix

### A.1. Phase Shifts and Cross-Section in the Scattering of an Ahronov–Bohm Flux

The scattering of an electron on an edge dislocation is analogous to the scattering off a Aharonov–Bohm flux, *α*, [48]. An incoming plane wave of wavevector $\vec{k}$:

$$e^{-ikr\cos\phi }=\sum_{m}i^{m}e^{im\phi }{\left(-1\right)}^{m}J_{m}(kr)$$ (A1)

where *ϕ* = *θ_r_* *−θ_k_* is the angle between the direction of propagation and the one in real space, is scattered by the A-B flux located at the origin of the frame. A solution of the Schrödinger equation:

$$\left[\frac{\partial^{2}}{\partial r^{2}}+\frac{1}{r}\frac{\partial }{\partial r}+\frac{1}{r^{2}}{\left(\frac{\partial }{\partial \phi }+i\alpha \right)}^{2}+k^{2}\right]\psi \left(r\right)=0$$ (A2)

is:

$$\psi \left(r\right)=\sum_{m=-\infty }^{\infty }a_{m}e^{im\phi }J_{\left|m+\alpha \right|}\left(kr\right)$$ (A3)

When fixing the boundary conditions far away from the scattering center, we use the asymptotic expansion of the Bessel functions, *J_ν_*(*kr*). Prior to the scattering event, we have to match the incoming circular wave with the incoming asymptotic circular component of the solution:

$$\text{incoming circular wave }\sim \frac{1}{2}\sqrt{\frac{2}{\pi kr}}\sum_{m=-\infty }^{\infty }{\left(-i\right)}^{m}e^{im\phi }e^{-ikr}e^{im\frac{\pi }{2}}e^{i\frac{\pi }{4}}$$ (A4)

$$\text{incoming circular component of }\psi \sim \frac{1}{2}\sqrt{\frac{2}{\pi kr}}\sum_{m=-\infty }^{\infty }a_{m}e^{im\phi }e^{-ikr}e^{i|m+\alpha |\frac{\pi }{2}}e^{i\frac{\pi }{4}}e^{i\alpha \theta_{k}}$$ (A5)

The matching requires that: $a_{m}=e^{-i|m+\alpha |\pi /2}e^{-i\alpha \theta_{k}}$. The same for the outgoing waves:

$$\text{outgoing circular wave }\sim \frac{1}{2}\sqrt{\frac{2}{\pi kr}}\sum_{m=-\infty }^{\infty }{\left(-i\right)}^{m}e^{im\phi }e^{ikr}e^{-im\pi /2}e^{-i\pi /4}+f\left(\phi \right)\frac{e^{ikr}}{\sqrt{r}}$$ (A6)

$$\text{outgoing circular component of }\psi \sim \frac{1}{2}\sqrt{\frac{2}{\pi kr}}\sum_{m=-\infty }^{\infty }e^{im\phi }e^{ikr}e^{-2i|m+\alpha |\pi /2}e^{-i\pi /4}e^{-i\alpha \theta_{k}}$$ (A7)

The outgoing wave is the superposition of the unscattered wave and of the one diffused with scattering amplitude *f*(*ϕ*). The phase shift in the scattering is obtained by comparing the two outgoing plane waves of Equations (A6) and (A7):

$$\delta_{m}=-\frac{\pi }{2}(|m+\alpha |-|m|)$$ (A8)

The scattering amplitude can be identified in terms of the phase shift as:

$$f\left(\phi \right)=\sqrt{\frac{1}{2\pi k}}\sum_{m=-\infty }^{\infty }{\left(-1\right)}^{m}\left[e^{2i\delta m}-1\right]\;e^{im\phi }$$ (A9)

### A.2. Green’s Function for Dirac Electrons Scattered by an External Aharonov–Bohm Flux

#### A.2.1. Green’s Function for Free Dirac Electrons

We first address the Green’s function for Dirac electrons propagating freely in two dimensions (2D). The Green’s function for the complete 2D Dirac operator, which includes the time dependence, satisfies the defining equation:

$$[\gamma^{0}\partial_{t}+\gamma^{i}\partial_{i}]G_{0}(x,x^{\prime })=-i\delta (x,x^{\prime })Ι$$ (A10)

where $x\equiv (\vec{r},t)$ and γ^0^ = −*iσ_z_*, γ^1^ = −*iσ_y_* γ^2^ = −*iσ_x_*. The Fourier transform, $G_{0}(\vec{r},{\vec{r}}^{\prime };\omega )$, defined by:

$$G_{0}(x,x^{\prime })=\int d\omega \;e^{-i\omega (t-t^{\prime })}G_{0}(\vec{r},{\vec{r}}^{\prime };\omega )$$ (A11)

satisfies:

$$[-\sigma_{z}\omega +\gamma^{i}\partial_{i}]G_{0}(\vec{r},{\vec{r}}^{\prime };\omega )=-i\delta (\vec{r}-{\vec{r}}^{\prime })ΙΙ$$ (A12)

according to Equation (A10). Multiplying both sides of the equation by *σ_z_*, we obtain:

$$[\omega +i\sigma \cdot \nabla ]G_{0}(r,r^{\prime };\omega )=[\omega -H]G_{0}(\vec{r},{\vec{r}}^{\prime };\omega )=i\delta (\vec{r}-{\vec{r}}^{\prime })\sigma_{z}$$ (A13)

In the plane wave representation and circular coordinates, we obtain:

$$G_{0}(\rho ,\theta_{\rho };\omega )=-\frac{1}{{\left(\pi \hbar \upsilon_{F}\right)}^{2}}\int d^{2}k\;e^{i\vec{k}\cdot \vec{\rho }}\frac{\omega 1+\vec{k}\cdot \vec{\sigma }}{\omega^{2}-k^{2}}=-\frac{1}{{\left(\pi \hbar \upsilon_{F}\right)}^{2}}\int d^{2}k\;e^{i\vec{k}\cdot \vec{\rho }}\frac{1}{\omega^{2}-k^{2}}\left(\begin{array}{cc} \omega & k\;e^{-i\theta_{k}}\\ k\;e^{i\theta_{k}} & \omega \end{array}\right)$$ (A14)

where $\rho =|r-r^{\prime }|=\sqrt{r^{2}+{r^{\prime }}^{2}-2rr^{\prime }\;\text{cos}\left(\theta_{r}-\theta_{r^{\prime }}\right)}$ and *θ_ρ_* is the corresponding angle. The prefactor guarantees the equality with the *δ−*function at the right-hand side. The integration over the angle, *θ_k_*, of the matrix elements of Equation (A14) gives:

$$G_{0}(\rho ,\theta_{\rho };\omega )=-\frac{2\pi \omega }{{\left(\pi \hbar \upsilon_{F}\right)}^{2}}\int \frac{kdl}{\omega^{2}-k^{2}}\left(\begin{array}{cc} J_{0}(k\rho ) & -iJ_{-1}(k\rho )\;e^{-i\theta \rho }\\ iJ_{1}(k\rho )e^{i\theta_{\rho }} & J_{0}(k\rho ) \end{array}\right)$$ (A15)

The integral over the modulus of *k* is more cumbersome. When applying the residue theorem, the circuit is closed at infinity in the complex *p−*plane. The contribution at infinity vanishes. As the poles are on the real axis, just half of the poles has to be included. The integrals on the real axis are of the kind:

∫0∞pdpJv(pρ)ω2−p2→12ω[∫0∞pdpω−pJv(pρ)+∫0∞pdpω+pJv(pρ)]=12ω[∫0∞J v(pρ)−(−1)1−v∫−∞0Jv(pρ)]pdpω−p (A16)

where *ν* = 0*,* 1. *J*_0_(*pρ*) is even with respect to the change *p → −p*. Hence, the *ν* = 0 integral is extended to the full real axis, *p* *∈* (*−∞,* +*∞*), and just the half pole survives as an exact result. The *ν* = 1 integral cannot be dealt with like that, because *J*_1_(*pρ*) is odd. There is a contribution coming from the integration along the imaginary axis that should be evaluated. However, as this contribution is exponentially convergent far from the defect center, we shall drop it and keep just the half pole also for the *ν* = 1 Bessel function. This choice corresponds to a semiclassical saddle point approximation in a path-integral formalism [95,96]. Which one of the two poles, *ω* = *sp* (*p >* 0), is contributing depends on the the sign of *ω*. In conclusion, the approximated result for *r > r′* is:

$$G_{0}(\rho ,\theta_{\rho };\omega )=-\frac{i\omega }{{\left(\hbar \upsilon_{F}\right)}^{2}}\left(\begin{array}{cc} J_{0}(\omega \rho ) & -isJ_{-1}(\omega \rho )\;e^{-i\theta_{\rho }}\\ isJ_{1}(\omega \rho )\;e^{i\theta_{\rho }} & J_{0}(\omega \rho ) \end{array}\right),\;\;\;s=sign\{\omega \}$$ (A17)

This result is asymptotically sound. The density of state (including spin degeneracy) is:

$$-\frac{1}{2\pi }\text{tr}\int d^{2}Rℑm\{G_{0}(\rho =0;\omega )\}=\frac{\omega }{\pi {\left(\hbar \upsilon_{F}\right)}^{2}}Α$$ (A18)

where *A* is the area. This is the correct result. It can be shown that Equation (A17) satisfies Equation (A13). The asymptotic expansion of the Bessel functions with appropriate causality conditions yields [97]:

$$\underset{\rho \to \infty }{\lim}G_{0}(\rho ,\theta_{\rho };\omega )\sim -i\frac{e^{i(\omega \rho -\pi /4)}}{{\left(\hbar \upsilon_{F}\right)}^{2}}\sqrt{\frac{2\pi |\omega |}{\rho }}\left(\begin{array}{cc} 1 & s\;e^{-i\theta_{\rho }}\\ s\;e^{i\theta_{\rho }} & 1 \end{array}\right)$$ (A19)

The spectral representation is also useful. Retaining just the half pole contribution, again, we have for *r > r′*:

$$G_{0}(\vec{r},{\vec{r}}^{\prime },\omega )=-i\frac{\omega }{{\left(\hbar \upsilon_{F}\right)}^{2}}\sum_{m}e^{im(\theta_{r}-\theta_{r^{\prime }})}\left(\begin{array}{cc} J_{m}(\omega r)\;J_{m}^{*}(\omega r^{\prime }) & -isJ_{m}(\omega r)J_{m+1}^{*}(\omega r^{\prime })\;e^{-i\theta_{r^{\prime }}}\\ -is\;J_{m+1}(\omega r)J_{m}^{*}(\omega r^{\prime })e^{i\theta_{r}} & -J_{m+1}(\omega r)\;J_{m+1}^{*}\;(\omega r^{\prime })e^{i(\theta_{r}-\theta_{r^{\prime }})} \end{array}\right)$$ (A20)

The Bessel functions, being of integer order, are real. However, by keeping the star in the notation, we intend to remind that, when *r < r′*, one has to exchange *r*↔r′ and *θ_r_* *↔ −θ_r′_*. The series could be resumed by using Graf’s theorem, to recover Equation (A17).

#### A.2.2. Green’s Function with the Aharonov-Bohm Flux

The wavefunction in the presence of an A-B flux can be expressed through a Fourier series (*ϕ* = *θ_r_−θ_p_* is the angle between $\vec{p}$ and $\vec{r}$) as:

$$\Psi_{s}(r,\theta_{r})=\sum_{m}e^{im\phi }\frac{1}{\sqrt{2}}\left(\begin{array}{c} u_{m}\\ s\;\upsilon_{m}\;e^{i\theta_{r}} \end{array}\right)$$ (A21)

where the functions, *u_m_*(*r*)*, v_m_*(*r*), solve the equation of an A-B flux line *α* at the origin (*ħv_F_* = 1), given by Equation (A2):

$$\left[\frac{\partial^{2}}{\partial r^{2}}+\frac{1}{r}\frac{\partial }{\partial r}+\frac{1}{r^{2}}{\left(\frac{\partial }{\partial \phi }+i\alpha \right)}^{2}+p^{2}\right]\psi (r)=0$$ (A22)

They are:

$$\begin{array}{l} u_{m}(r)=\int d\phi e^{i[pr\;\cos\phi -(m+\alpha )\phi -\alpha \theta_{\rho }]}={\left(-i\right)}^{m+\alpha }e^{-i\alpha \theta_{\rho }}J_{m+\alpha }(pr)\\ \upsilon_{m}(r)=\int d\phi \;e^{i[pr\;\cos\phi -(m+1+\alpha )\phi -\alpha \theta_{\rho }}={\left(-i\right)}^{m+1+\alpha }e^{-i\alpha \theta_{\rho }}J_{m+1+\alpha }(pr) \end{array}$$ (A23)

Green’s function is (*s* = *sign*{ω}):

$$G\;(\vec{r},{\vec{r}}^{\prime },\omega )=-\frac{1}{{\left(\pi \hbar \upsilon_{F}\right)}^{2}}\;\int d\vec{p}\;{\sum_{m,m^{\prime },s}\frac{e^{i(m\phi_{r}-m^{\prime }\phi_{r^{\prime }})}}{\omega -sp}\frac{1}{2}\left(\begin{array}{c} u_{m}^{\mathrm{out}}(r)\\ s\;\upsilon_{m}^{\mathrm{out}}(r)\;e^{i\theta_{r}} \end{array}\right)}^{\left(\upsilon_{m^{\prime }}^{in}{}^{\;*}(r^{\prime })\;\;s\;\;\upsilon_{m^{\prime }}^{in}{}^{\;*}(r^{\prime })\;e^{-i\theta_{r^{\prime }}}\right)}$$ (A24)

By imposing scattering causal conditions for *r > r*′, we take outgoing waves, *u^out^*(*υ^out^*) *∼* *J_m_*_+0(1)+_*_α_*(*pr*), and incoming waves, *u^in^*(*υ^in^*) *∼* *J_m_*_+0(1)_(*pr*′).

Furthermore, in this case, Green’s function allows for the trivial integration of the angle, *θ_p_*, because nothing depends on the angle, *θ_p_*. We get, for *r > r*′:

$$G\;(\vec{r},{\vec{r}}^{\prime },\omega )=-\frac{{\left(-i\right)}^{\alpha }}{{\left(\pi \hbar \upsilon_{F}\right)}^{2}}\;\int_{0}^{\infty }pdp\sum_{m,s}\frac{e^{im(\theta r-\theta_{r^{\prime }})}}{\omega -sp}\frac{1}{2}\left(\begin{array}{cc} J_{m+\alpha }(pr)J_{m}^{*}(pr^{\prime }) & is\;J_{m+\alpha }(pr)\;J_{m+1}^{*}(pr^{\prime })\;e^{-i\theta_{r^{\prime }}}\\ -is\;J_{m+1+\alpha }(pr)J_{m}^{*}(pr^{\prime })\;e^{i\theta_{r}} & J_{m+1+\alpha }(pr)\;J_{m+1}^{*}(pr^{\prime })\;e^{i(\theta_{r}-\theta_{r^{\prime }})} \end{array}\right)$$ (A25)

Again, we can apply the Graf summation theorem with the condition *r > r′*:

$$J_{v}(\omega \rho )e^{iv\chi }=\sum_{m=-\infty }^{{}^{+\infty }}J_{m+v}(\omega r)\;J_{m}(\omega r^{\prime })\;e^{im(\theta -\theta^{\prime })}$$ (A26)

to get:

$$G\;(\vec{r},{\vec{r}}^{\prime },\omega )=-\frac{{\left(-i\right)}^{\alpha }}{2{\left(\pi \hbar \upsilon_{F}\right)}^{2}}\;e^{i\alpha (\theta -\theta^{\prime })/2}\sum_{s}\int_{0}^{\infty }\frac{pdp}{\omega -sp}\left(\begin{array}{cc} J_{\alpha }(p\rho ) & -s\;e^{-i(\theta -\theta^{\prime })}\;e^{-i\theta_{\rho }}J_{\alpha -1}(p\rho )\\ -s\;e^{i\theta_{\rho }}e^{i(\theta -\theta^{\prime })}J_{1+\alpha (p\rho )} & J_{\alpha }(p\rho ) \end{array}\right)$$ (A27)

where we have used the approximation *θ_ρ_* *≈* *π* + (*θ* + *θ′*)*/*2. When *r < r′*, we have to exchange *r ↔ r′* and *θ_r_* *↔ −θ_r′_*.

For the scattering of a central potential, outgoing spherical waves for *ω >* 0 imply that $J_{v}(z)\to H_{v}^{\left(1\right)}(z)$, and we can use the property $H_{-v}^{\left(1\right)}(z)=e^{v\pi i}H_{v}^{\left(1\right)}(z)$. However, for *z →* 0, $H_{v}^{\left(1\right)}(z)$ has a cut on the negative real axis with a branch point at zero. The circuit is depicted in Figure A1. The contribution of the quarter of the circle around the branch point at the origin vanishes for *ω* ≠ 0. We shall drop the integral and keep just the half pole with *s* = *sign*{ω} also in this case. Note that the propagation of holes (*ω* < 0) requires that $H_{v}^{\left(1\right)}(z)$ is substituted with $H_{v}^{\left(2\right)}(z)$.

### A.3. Simplified Derivation of Boundary States of a TI in the Long Wavelength Approximation

We can exhibit a simple analytical form of the electronic wavefunctions of a two-band TI in the long wavelength limit, which is described by the model Hamiltonian of Equation (58) (dropping *h*_0_*−μ*) [56], provided we make a few simplifying assumptions. To obtain localized states at the boundary, the surface is assumed to be flat in the *z* = 0 plane, so that we can Fourier transform with respect to the coordinates parallel to the surface. We put the chemical potential *μ* = 0 and drop all the second order space derivatives appearing in $ℳ=M+C_{1}\;\partial_{z}^{2}+C_{2}\partial_{\left|\right|}^{2}$, for simplicity. The latter is a delicate point, as the relative signs of *M, C_1_, C_2_* qualify the material as topologically trivial or non-trivial and allow the boundary wavefunctions to drop to zero inside the surface. However the model does not loose its potential if the bulk gap, *M*, is taken to change sign at the boundary. We can interpret the change of sign of *M* as the moving from a topologically trivial side to the topologically non-trivial side. Boundary states will display their maximum at *z* = 0 and decay exponentially away from both sides of the matching surface. In addition, just the amplitude of the wavefunction has to be matched at *z* = 0, because the Hamiltonian is first order in the derivatives. We will adopt these simplifications, which allow us to write down the localized solutions in a closed form, quite easily.

The two bands have opposite parity. The toy model Hamiltonian, close to the Γ point, derived from Equation (58), takes the form:

$${ℋ}_{0}\;[k_{\left|\right|},z]=(\psi_{g↑}^{†}\;\psi_{u↑}^{†}\;\psi_{g↓}^{†}\;\psi_{u↓}^{†})\;\left(\begin{array}{cccc} M & -i\partial_{z} & 0 & k_{-}\\ -i\partial_{z} & -M & k_{-} & 0\\ 0 & k_{+} & M & i\partial_{z}\\ k_{+} & 0 & i\partial_{z} & -M \end{array}\right)\;\left(\begin{array}{c} \psi_{g↑}\\ \psi_{g↑}\\ \psi_{g↑}\\ \psi_{g↑} \end{array}\right)$$ (A28)

Deep in the bulk, for $\vec{k}\to -\vec{k}$, the field, $\psi_{g\sigma }(\vec{k})$, is even, while the field, $\psi_{u\sigma }(\vec{k})$, is odd. The bulk eigenfunctions correspond to the eigenvalues $E=\pm \sqrt{M^{2}+k^{2}+k_{z}^{2}}$, where $k^{2}=k_{x}^{2}+k_{y}^{2}=k_{+}k_{-}$ with $k_{\pm }=k_{x}\pm ik_{y}$. They are:

$$|E,\vec{k},1\rangle =\frac{1}{\sqrt{N}}\left(\begin{array}{c} E+M\\ k_{z}\\ 0\\ k_{+} \end{array}\right)\;e^{ik_{z}z}e^{ik_{\left|\right|}\cdot r_{\left|\right|}},|E,\vec{k},2\rangle =\frac{1}{\sqrt{N}}\left(\begin{array}{c} 0\\ k_{-}\\ E+M\\ -k_{z} \end{array}\right)\;e^{ik_{z}z}e^{ik_{\left|\right|}\cdot r_{\left|\right|}}$$ (A29)

where *N* is the normalization.

Localized states at the surface could be:

$$\begin{array}{c} |1+;k_{\left|\right|}\rangle =\frac{1}{\sqrt{N}}\left(\begin{array}{c} M+E\\ iM\;sign(z)\\ 0\\ k_{+} \end{array}\right)\;e^{-M|z|}e^{ik_{\left|\right|}\cdot r_{\left|\right|}},\;with\;eigenvalue\;E=k\\ |2+;k_{\left|\right|}\rangle =\frac{1}{\sqrt{N}}\left(\begin{array}{c} 0\\ k_{-}\\ M+E\\ -iM\;sign(z) \end{array}\right)e^{-M|z|}e^{ik_{\left|\right|}\cdot r_{\left|\right|}}\;with\;eigenvalue\;E=k\\ |1-;k_{\left|\right|}\rangle =\frac{1}{\sqrt{N}}\left(\begin{array}{c} M+E\\ iM\;sign(z)\\ 0\\ k_{+} \end{array}\right)e^{-M|z|}e^{ik_{\left|\right|}\cdot r_{\left|\right|}},\;with\;eigenvalue\;E=-k\\ |2-;k_{\left|\right|}\rangle =\frac{1}{\sqrt{N}}\left(\begin{array}{c} 0\\ k_{-}\\ M+E\\ -iM\;sign(z) \end{array}\right)e^{-M|z|}e^{ik_{\left|\right|}\cdot r_{\left|\right|}},\;with\;eigenvalue\;E=-k \end{array}$$ (A30)

However, so as they stand, the wavefunctions are not continuous at *z* = 0. We have to implement continuity at *z* = 0 to obtain physical states. Using the time reversal operator, Θ, and the parity operator, $P:\{\vec{r}\to -\vec{r}\}$, we note that:

$$P\Theta H\Theta^{†}P^{†}=-H(-M)$$ (A31)

Therefore *P*Θ*H*Θ^†^*P*^†^ is a good Hamiltonian for the other half space, *z → −z*, when *M → −M*. However, the minus sign on the right-hand side of Equation (A31) implies that the two eigenvalues are exchanged. Then, the matching condition is:

$$\begin{array}{ccc} {A\;|1+;k_{\left|\right|}\rangle +B|2+;k_{\left|\right|}\rangle |}_{z=0} & ={C\;|P\Theta 1-;k_{\left|\right|}\rangle +D\;|P\Theta 2-;k_{\left|\right|}\rangle |}_{z=0} & for\;E=k \end{array}$$ (A32)

$$\begin{array}{ccc} {A^{\prime }\;|1-;k_{\left|\right|}\rangle +\;B^{\prime }|2-;k_{\left|\right|}\rangle |}_{z=0} & =C^{\prime }\;{|P\Theta 1+;k_{\left|\right|}\rangle +D^{\prime }\;|P\Theta 2+;k_{\left|\right|}\rangle |}_{z=0} & for\;E=-k \end{array}$$ (A33)

Apart from a factor *e^ikk·rk^*, Equation (A32) for *E* = *k*, becomes:

$$\begin{array}{l} {A\left(\begin{array}{c} M+k\\ iM\\ 0\\ k_{+} \end{array}\right)\;e^{-Mz}+B\left(\begin{array}{c} 0\\ k_{-}\\ M+k\\ -iM \end{array}\right)\;e^{-Mz}|}_{z=0^{+}}\\ {C\left(\begin{array}{c} 0\\ -k_{-}\\ -M-k\\ iM \end{array}\right)\;e^{Mz}+D\left(\begin{array}{c} M+k\\ iM\\ 0\\ k_{+} \end{array}\right)\;e^{Mz}|}_{z=0^{-}} \end{array}$$ (A34)

As the spinor with B is opposite to the one with C, the determinant vanishes, and the solution exists. By inspection, we find: *A = D* = 1, *B = −C* = 1, so that:

|sur​f,+;k∥〉∝(M+kk−+iM M+kk+−iM)e−M|z|eik∥⋅r∥,forE=+k (A35)

By exchanging *k ↔ −k*, we obtain the solution for *E = −k*:

$$|\mathrm{sur}\text{​}f,-;k_{∥}\rangle \propto \left(\begin{array}{c} M-k\\ k_{-}+iM\\ M-k\\ k_{+}-iM \end{array}\right)e^{-M|z|}e^{ik_{∥}\cdot r_{∥}},\;for\;E=-k$$ (A36)

Note that, at the neutrality point (*E* = 0), the degenerate zero energy states have wavefunctions:

$$|\mathrm{sur}\text{​}f,\to ;0\rangle \propto \left(\begin{array}{c} 1\\ i\\ -1\\ i \end{array}\right)e^{-M|z|},\;|sur\text{​}f,\leftarrow ;0\rangle \propto \left(\begin{array}{c} 1\\ i\\ 1\\ -i \end{array}\right)e^{-M|z|},\;for\;E=0$$ (A37)

They are time reversed states.
